# The Effect of Hexavalent Chromium on the Incidence and Mortality of Human Cancers: A Meta-Analysis Based on Published Epidemiological Cohort Studies

**DOI:** 10.3389/fonc.2019.00024

**Published:** 2019-02-04

**Authors:** Yujiao Deng, Meng Wang, Tian Tian, Shuai Lin, Peng Xu, Linghui Zhou, Cong Dai, Qian Hao, Ying Wu, Zhen Zhai, Yue Zhu, Guihua Zhuang, Zhijun Dai

**Affiliations:** ^1^Department of Breast Surgery, Guangzhou Women and Children's Medical Center, Guangzhou Medical University, Guangzhou, China; ^2^Department of Oncology, The Second Affiliated Hospital of Xi'an Jiaotong University, Xi'an, China; ^3^Department of Epidemiology and Biostatistics, Xi'an Jiaotong University Health Science Center, School of Public Health, Xi'an, China

**Keywords:** hexavalent chromium, cancer, mortality, incidence, meta-analysis

## Abstract

**Background:** Hexavalent chromium [Cr(VI)] is an occupational carcinogen that can cause lung and nasal cancers, but its association with mortality and incidence in many other cancers is unclear.

**Objectives:** In this meta-analysis, we aimed to evaluate the relationship between exposure to Cr(VI) and the mortality and incidence of human cancers.

**Methods:** We performed a search of the literature and extracted the standardized mortality ratios (SMRs), standardized incidence ratios (SIRs), and their corresponding 95% confidence intervals (CIs), to estimate risk values. Subgroup analyses were conducted by sex, occupation, and types of cancer to identify groups that were at high-risk or predisposed to certain cancers.

**Results:** A total of 47 cohort studies covering the period 1985–2016 were included (37 studies reporting SMRs and 16 studies reporting SIRs). The summary SMR for all studies combined was 1.07 (95% CI: 1.01–1.15). Summary SMRs were higher among chromate production workers, chrome platers, and masons, and especially male workers. In the subgroup analysis, Cr(VI) exposure was related to a higher risk of death owing to lung, larynx, bladder, kidney, testicular, bone, and thyroid cancer. The meta-SIR of all studies combined was 1.06 (95% CI: 1.04–1.09). Summary SIRs were elevated among cement industry workers and tanners. Cr(VI) exposure was related to an elevated risk of respiratory system, buccal cavity, pharynx, prostate, and stomach cancers.

**Conclusions:** Cr(VI) might cause cancers of the respiratory system, buccal cavity and pharynx, prostate, and stomach in humans, and it is related to increased risk of overall mortality owing to lung, larynx, bladder, kidney, testicular, bone, and thyroid cancer. In addition, there was a strong association between incidence and mortality risk of cancers and concentration of Cr(VI) in the air and the exposure time.

## Introduction

### Rationale

Occupation-related cancers are an important public health issue with serious socioeconomic effects. It is essential to recognize and identify occupational carcinogens for risk prevention, surveillance, and compensation of exposed workers. According to data from the International Agency for Research on Cancer (IARC), hexavalent chromium [Cr(VI)] has been classified as a Group I occupational carcinogen. In addition, there is sufficient evidence that Cr(VI) is related to cancers of the lung, nasal cavity, and paranasal sinuses (1). The European Commission conducted a socioeconomic, health, and environmental impact assessment and found that strong factors related to attributable cancer deaths include hardwood dust, Cr(VI), and respirable crystalline silica (2). Cr(VI) exposure exists in many industries, including chromate production, stainless steel production, welding, chrome pigment production, chrome plating, tanning, cement production, and aircraft manufacturing. Workers are often exposed to Cr(VI) by inhalation and dermal contact (3). A meta-analysis performed by Welling et al. suggested that Cr(VI) is a stomach carcinogen in humans (4). Hara et al. found that Cr(VI) exposure may increase the risk of brain cancer and malignant lymphoma (5). Iaia et al. confirmed that exposure to Cr(VI) increased lung, bladder, and pancreatic cancer mortality among tanners and found that Cr(VI) increased mortality due to myeloid leukemia and tumors of the endocrine glands (6).

Great progress has been made in identifying the correlation between exposure to Cr(VI) and some respiratory cancers. Nevertheless, there is an ongoing need for research on the relationship between Cr(VI) and other cancers, which has not been evaluated owing to inadequate epidemiological evidence and a paucity of quantitative exposure data.

### Research Question

Does Cr(VI) pose a risk of increased cancer incidence and mortality in populations with high exposure to Cr(VI)?

## Materials and Methods

This study was conducted following the 2015 Preferred Reporting Items for Systematic Review and Meta-Analysis Protocols (PRISMA-P) and the Meta-Analysis of Observational Studies in Epidemiology (MOOSE) guidelines (7, 8), which was shown in Supplemental Table 4.

### Search Strategy

Databases (PubMed, EMBASE, Web of Science, the Cochrane Library, Google Scholar, CNKI, WANFANG, and VIP) were searched by two authors independently to obtain all related epidemiological data up to May 28, 2018. The data covered the period 1985–2016. The search strategies used combinations of the following keywords and phrases: hexavalent chromium, chromium(VI), Cr(VI), chromate, chrome, chromate production, stainless steel, welding, chrome pigment production, chrome plating, ferrochrome production, leather, tanning, tanners, cement, concrete, metal plating, cancer, neoplasia, neoplasm, tumors, malignant neoplasms, malignancy, cohort study, cancer mortality, cancer incidence, cancer morbidity, standardized mortality ratio (SMR), standardized incidence ratio (SIR), with “AND” and “OR” used to narrow the range of articles identified. In addition, we searched all studies in the reference lists of published reviews and all relevant meta-analyses.

### Selection Criteria

All contents of the candidate articles and data were read, and the data were extracted by two authors (DYJ and WM) independently. Publications included in this meta-analysis met the following criteria: (1) the exposed population and study region was stated; (2) the exposure factor was clear and exposure was to Cr(VI); (3) the cancer mortality or incidence data were included; (4) the study was a cohort study; (5) the exposure time and dose were declared; (6) a follow-up period was included; (7) the SMR or SIR with confidence intervals (CIs) were listed; (8) for studies with different latency periods, the SMR/SIR for the longest period was selected; (9) for studies with different exposure levels, the result of the highest level was selected.

The exclusion criteria were as follows: (1) unavailable data (no CIs of SMR or SIR, or only data of relative risk, odds ratio, proportional mortality ratio, or hazard ratio); (2) duplicated data; for overlapping populations, only the largest number of cases or most recent data were selected; (3) meta-analysis study, case report, review, or letter; (4) occupational exposure to materials other than Cr(VI), such as asbestos or nickel;(5) unpublished data, including government reports; (6) professions such as shoemaking (non-leather) or general building work; (7) exposure to Cr(VI) in drinking water.

### Quality Assessment

Each study was assigned a quality score based on the Newcastle-Ottawa assessment scale (NOS) (9), by two authors independently, to ensure the research quality. Any disagreements were discussed among the group members until consensus was reached. NOS scores range from 0 to 9; the NOS score of the studies included in this meta-analysis ranged from 6 to 8.

### Data Extraction

The following data were extracted by two authors independently: first author' name, publication year, country in which the study was conducted, sex of the study population, exposure time, follow-up period, cancer incidence rate, cancer types, occupations, number of cancer-specific deaths, study design, outcome indicator, and standardized mortality ratios (SMRs) or standardized incidence ratios (SIRs) with their 95% CIs.

### Statistical Analysis

The meta-SMRs and SIRs with their 95% CIs were calculated using either a fixed or random-effects model to evaluate the mortality and incidence of human cancers. The Cochran Q test and *I*^2^ statistic were used to assess heterogeneity. Heterogeneity was considered significant with *P* < 0.10 or *I*^2^ > 50%. The random-effects model was used for values of *I*^2^ > 50%, and the fixed-effects model was applied otherwise. Subgroup analyses were carried out according to geographical region, sex, cancer type, and profession, to screen susceptible populations and high-risk diseases. Begg's funnel plot and Egger's test were used to estimate publication bias (10, 11). Asymmetry in the funnel plot exists with a value of *P* < 0.05 in the Egger's test, indicating significant publication bias. A sensitivity analysis including more than 10 studies was conducted, to assess the individual effect of each study on the results. All statistical tests were two-sided, with *P* < 0.05 indicating statistical significance. Stata version.14.0 (Stata Corp LL, College Station, TX, USA) was used to analyze the data.

## Results

### Study Selection and Characteristics

**Figure 1:**
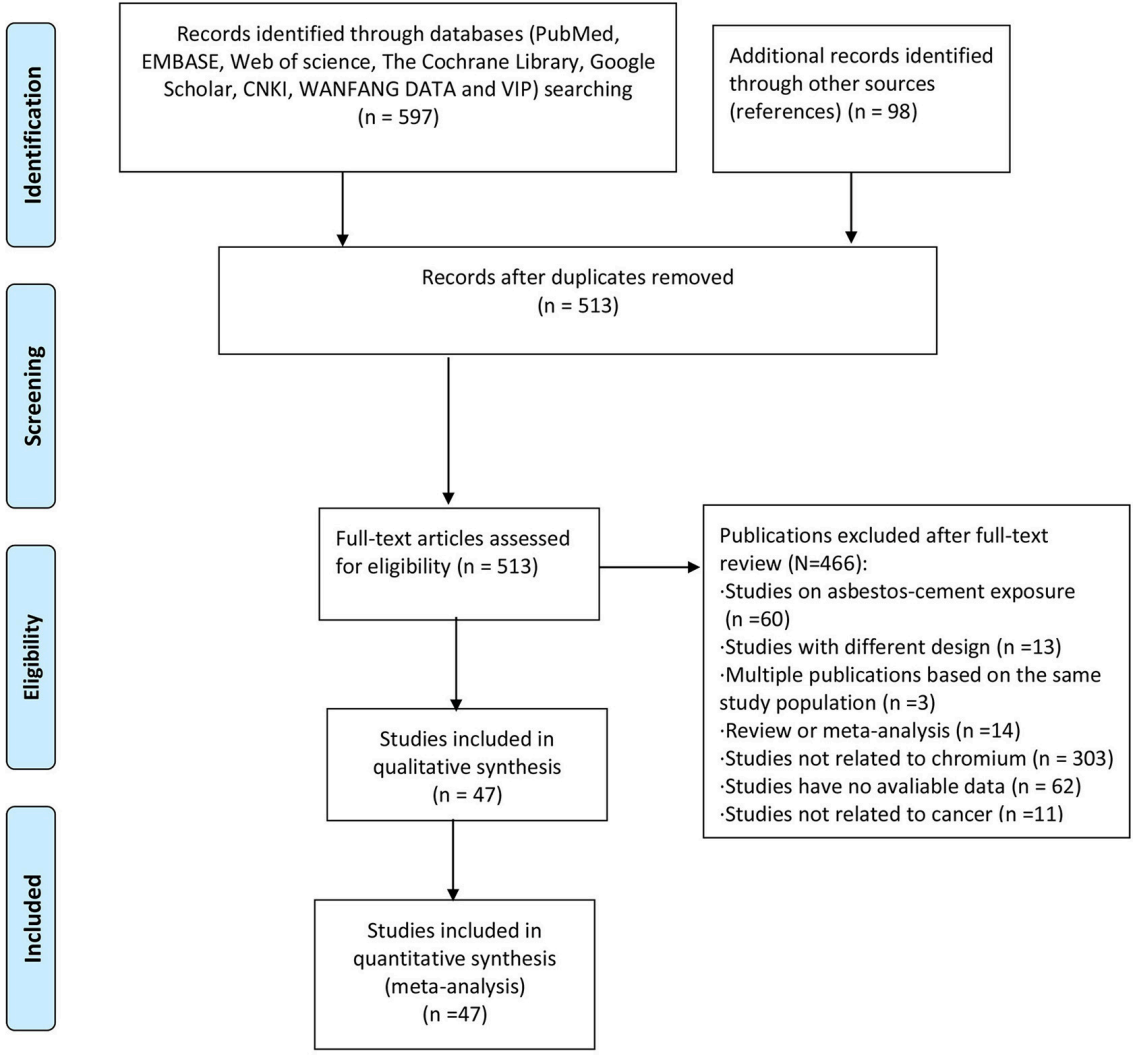
Flow chart of the study procedure.

Of nearly 700 entries initially identified in the database search, only 512 articles were included after duplicates were removed and full texts were read carefully. The specific screening procedures are listed in the flow chart (Figure 1). We ultimately included 37 cohorts with SMRs reported and 16 with SIRs reported (retrospective or historical prospective) from 47 separate studies. The total number of cases was 1,141,094, ranging from 198 to 892,591 cases per study. Included studies covered 14 countries including the United States, Unite Kingdom, Finland, France, Korea, Japan, Germany, Italy, Lithuania, Sweden, Switzerland, Iceland, Denmark, and Norway. The study populations included both male and female workers who were occupationally exposed to Cr(VI), such as chromate production workers, cement industry workers, stainless steel welders, chrome platers, aircraft manufacturing workers, tanners, painters, and masons. In most studies, the exposure time to Cr(VI) was more than 1 year. Key characteristics of the included studies are summarized in Tables 1, 2. All the results of subgroup analysis were shown in Supplementary Tables 1, 2. Studies excluded from this meta-analysis and the reasons for exclusion are shown in Supplementary Table 3.

**Table 1:** Characteristics and results of SMR studies included in the meta-analysis.

**Study**	**Country**	**District**	**Sex**	**No of cases**	**Cancer deaths (Observed)**	**Exposure time(year)**	**Follow-up time**	**Cancer type**	**Occupation**	**Effect measure**	**SMR**	**95% CI**		**NOS score**
												**LL**	**UL**	
Proctor et al. (12)	USA	North America	male	714	167	>1	1940–2011, 34.4 (0.1–69.9) years, 24,535 person-years	Lung cancer	Chromate production workers	SMR	1.86	1.45	2.28	8
Proctor et al. (12)	USA	North America	male	198	37	1	1940–2011, 6,549 person-years	mixed	Chromate production workers	SMR	1.28	0.87	1.69	7
Gibb et al. (13)	USA	North America	male	2,354	460	6.4-57.2	1950–2011, 38.9 (+/−14.2) years, 91,186 person-years	mixed	Chromate production workers	SMR	1.16	1.06	1.27	8
Huvinen et al. (14)	Finland	Europe	mix	8,088	133	24.7	1971–2012, 199,760 person-years	mixed	Chromate production workers	SMR	0.88	0.73	1.03	8
Dab et al. (15)	France	Europe	mix	9,118	207	>1	1990–2005, 13.4 years, 122,124 person-years	mixed	Cement production workers	SMR	0.8	0.69	0.92	8
Koh et al. (16)	Korea	Asia	male	5,146	103	35.3(9.6)	1992–2007, 74,123 person-years	mixed	Cement production workers	SMR	0.83	0.68	1.01	7
Lipworth et al. (17)	USA	North America	mix	7,458	980	>1	1960–2008, 226,885 person-years, 31.8 years	mixed	Aircraft Manufacturing Workers	SMR	0.99	0.92	1.05	8
Hara et al. (5)	Japan	Asia	mix	1,193	97	>0.5	1976–2003, 27 years, 26,000 person-years	mixed	Chromium platers	SMR	1.05	0.86	1.27	8
Birk et al. (18)	Germany	Europe	mix	901	47	>1	1958–1998, 14,700 person-years	mixed	Chromate production workers	SMR	0.98	0.72	1.3	7
Iaia et al. (6)	Italy	Europe	mix	972	21	>1	1970–1998, 14,402 person-years	mixed	Tanners	SMR	0.66	0.44	0.95	7
Park et al. (19)	Korea	Asia	male	44,974	801	>1	1992–2001, 414,259 person-years,10 years	mixed	Welders	SMR	0.79	0.7	0.9	8
Park et al. (20)	USA	North America	male	2,357	122	3.1	1950–1992	Lung cancer	Chromium workers	SMR	1.78	1.5	2.11	6
Smailyte et al. (21)	Lithuania	Europe	male	2,498	121	>1	1978–2000, 43,490 person-years	mixed	Cement production workers	SMR	1.3	1	1.5	7
Stern et al. (22)	USA	North America	mix	9,352	548	>1	1940–1993, 265,499 person-years	mixed	Tanners	SMR	0.9	0.83	0.98	7
Luippold et al. (23)	USA	North America	mix	482	90	>1	1940–1997, 14,048 person-years	mixed	Chromate production workers	SMR	1.55	1.25	1.91	7
Steenland et al. (24)	USA	North America	male	4,459	258	>2 (8.5)	1988–1998	mixed	Welders	SMR	1.25	1.09	1.41	6
Moulin et al. (25)	France	Europe	mix	4,897	216	>1 (16.7)	1968–1992, 18.1 years, 87,247 person-years	mixed	Welders	SMR	0.97	0.85	1.11	8
Sorahan et al. (26)	UK	Europe	mix	1,087	131	>0.25	1972–1997	mixed	Chromium platers	SMR	1.35	1.12	1.62	6
Boice et al. (27)	USA	North America	mix	3,634	251	>1	1960–1996, 88,224 person-years (mean 24.2 years)	mixed	Aircraft Manufacturing Workers	SMR	0.93	0.82	1.06	8
Sorahan et al. (28)	UK	Europe	male	1,762	65	>0.5	1946–1995, 48135.1 person-years	Lung cancer	Chromium platers	SMR	1.25	0.93	1.66	7
Montanaro et al. (29)	Italy	Europe	mix	1,244	123	>0.5	1955–1994,36 414 person-years	mixed	Tanners	SMR	1.12	0.94	1.34	7
Jakobsson et al. (30)	Sweden	Europe	male	727	61	>1	1952–1993,15 years	mixed	Welders	SMR	0.8	0.6	1.1	7
Fu et al. (31)	UK	Europe	mix	4,215	646	>1	1939–1991, 10,3726 person-years	mixed	Tanners	SMR	0.75	0.69	0.81	7
Fu et al. (31)	Italy	Europe	mix	2,008	127	>1	1950–1990, 54,395 person-years	mixed	Tanners	SMR	1.16	0.96	1.38	7
Mikoczy, et al. (32)	Sweden	Europe	mix	2,060	119	>1	1952–1989	mixed	Tanners	SMR	1.09	0.91	1.31	6
Sorahan, et al. (33)	UK	Europe	male	10,438	1.129	>1 (9.3)	1946–1990, 29.2 years	mixed	Welders	SMR	1.19	1.12	1.26	7
Korallus, et al. 1(34)	Germany	Europe	male	1,417	62	>1	1982–1988, 12114.5 person-years	mixed	Chromate production workers	SMR	1.18	0.91	1.51	7
Korallus et al 2(34)	Germany	Europe	male	1,417	81	>1	1982–1988, 13868.2 person-years	mixed	Chromate production workers	SMR	1.58	1.27	1.94	7
Moulin et al. (35)	France	Europe	male	2,721	60	>1 (19.5)	1975–1988, 34,131 person-years	mixed	Welders	SMR	0.93	0.71	1.2	7
Jakobsson et al. (36)	Sweden	Europe	male	2,391	97	>1	1952–1986, 52,177 person-years	mixed	Cement production workers	SMR	0.83	0.68	1.02	7
Becker et al. (37)	Germany	Europe	mix	1,213	48	>0.5	1983–1988, 31,122 person-years	mixed	Welders	SMR	1.09	0.82	1.44	7
Steenland et al. (38)	USA	North America	male	4,459	105	>2	1974–1987,8.5years	mixed	Welders	SMR	1.02	0.83	1.24	7
Simonato et al. (39)	Europe	Europe	male	11,092	303	>1	1964–1984, 164,077 person-years	mixed	Welders	SMR	1.13	1	1.26	7
Moulin et al. (40)	France	Europe	male	2,269	27	>1	1952–1982	mixed	Welders	SMR	1.01	0.67	1.48	6
Horiguchi et al. (41)	Japan	Asia	male	265	7	>1	1965–1979, 3912.1 person-years	mixed	Chromium platers	SMR	1.13	0.45	2.32	7
Costantini et al. (42)	Italy	Europe	male	2,926	85	>0.5(12.3)	1950–1983	mixed	Tanners	SMR	0.97	0.77	1.19	6
Guberan et al. (43)	Switzerland	Europe	male	1,916	96	>1	1971–1984	mixed	Chromium platers	SMR	1.27	1.07	1.51	6
Svensson et al. (44)	Sweden	Europe	male	1,164	51	>0.25	1951–1983,24,624 person-years	mixed	Welders	SMR	0.98	0.74	1.3	7
Hayes et al. (45)	UK	Europe	male	1,879	101	>1	1940–1982, 50,724 person-years	mixed	Chromium workers	SMR	0.93	0.76	1.13	7
Rafnsson et.al. 1(46)	Iceland	Europe	male	449	26	20-30	1927–1982	mixed	Masons	SMR	1.45	0.95	2.13	6
Rafnsson et al. 2(46)	Iceland	Europe	male	389	21	20	1951–1982	mixed	Masons	SMR	1.64	1.02	2.51	6
Rafnsson et al. 3(46)	Iceland	Europe	male	251	17	30	1951–1982	mixed	Masons	SMR	2.23	1.3	3.57	6
Pippard et al. (47)	UK	Europe	male	260	30	>1	1939–1982	mixed	Tanners	SMR	1	0.68	1.43	6

**Table 2:** Characteristics and results of SIR studies included in the meta-analysis.

**Study**	**Country**	**District**	**Sex**	**No of cases**	**Cancer incidence (Observed)**	**Exposure time (year)**	**Follow-up time**	**Cancer type**	**Occupation**	**Effect measure**	**SIR**	**95% CI**		**NOS score**
												**LL**	**UL**	
Huvinen et al. (14)	Finland	Europe	Mix	8,146	408	5	1967–2011, 196,484 person-years	mixed	Welders	SIR	1.01	0.93	1.13	7
Koh et al. (16)	Korea	Asia	Male	5,596	174	33.1(9)	1992–2007, 47,233 person-years	mixed	Cement Industry Workers	SIR	1.01	0.87	1.18	6
Sorensen et al. (48)	Denmark	Europe	Mix	4,539	421	>1	1968–2003, 35 years, 125,762 person-years	mixed	Welders	SIR	1.02	0.93	1.13	8
Mikoczy et al. (49)	Sweden	Europe	Mix	2,027	351	>1	1958–1999, 56,022 person-years	mixed	Tanners	SIR	1.16	1.04	1.29	7
Smailyte et al. 1(21)	USA	North America	Mix	1,727	141	>1	1978–2000, 43,490 person-years	mixed	Cement Industry Workers	SIR	1.20	1.00	1.40	7
Smailyte et al. 2(21)	USA	North America	Mix	771	41	>1	1978–2000, 43,490 person-years	mixed	Cement Industry Workers	SIR	0.80	0.60	1.10	7
Knutsson et al. (50)	Sweden	Europe	Female	33,503	3572	>1	1971–1992,19.4 years, 582,225 person-years	mixed	Cement Industry Workers	SIR	1.07	1.03	1.10	8
Vasama-Neuvonen et al. (51)	Finland	Europe	Male	892,591	5072	>1	1971–1995, 15,481,680 person-years	Overian cancer	Chromium workers	SIR	0.70	0.40	1.20	7
Danielsen et al. (52)	Europe	Europe	Male	426	32	>10	1976–1992, 6,632 person-years	mixed	Welders	SIR	0.77	0.53	1.09	7
Rafnsson et al. (53)	Iceland	Europe	Male	1,172	148	>1	1955–1993	mixed	Cement Industry Workers	SIR	1.13	0.96	1.33	6
Jakobsson et al. (30)	Sweden	Europe	Male	719	112	>1	1958–1992	mixed	Welders	SIR	0.90	0.70	1.20	6
Danielsen et al. (54)	Norway	Europe	Mix	606	41	>1	1953–1992	mixed	Welders	SIR	1.00	0.71	1.35	6
Hansen et al. (55)	Denmark	Europe	Male	10,059	190	>1	1964–1986	mixed	Welders	SIR	0.94	0.81	1.08	6
Jakobsson et al. (36)	Sweden	Europe	Male	2,358	162	>1	1958–1986, 46,133 person-years	mixed	Cement Industry Workers	SIR	1.01	0.86	1.18	7
Simonato et al. (39)	Europe	Europe	Male	7510	363	>5	1964–1984, 98,376 person-years	mixed	Welders	SIR	1.20	1.08	1.33	7
Melkild et al. (56)	Norway	Europe	Male	783	252	>0.25	1946–1977	mixed	Welders	SIR	1.03	0.90	1.16	6
Svensson et al. (44)	Sweden	Europe	Male	1,164	84	>0.25	1958–1983, 20,936 person-years	mixed	Welders	SIR	1.03	0.82	1.27	7

*Mix, Male and Female; SIR, standardized incidence ratio; CI, confidence interval; LL, lower limit; UL, upper limit; NOS, The Newcastle-Ottawa quality assessment scale; mixed: Various cancer types; Europe (in country): Denmark, England, Finland, France, Germany, Italy, Norway, Scotland, Sweden*.

### Cancer Mortality

There were a total of 43 SMR studies comprising 167,397 cases and 8,277 cancer deaths. The aggregate data covered all 14 countries listed above and 8 types of occupation. All studies had follow-up periods of more than 5 years. The specific follow-up information was as follows: 5- to 10-year, 11- to 20-year, 21- to 30-year, 30- to 40-year, 40- to 50-year, and more than 50-year follow-up studies accounted for 11.60%, 16.30, 14.00, 23.30, 18.60, and 16.30% of studies, respectively.

**Figure 2:**
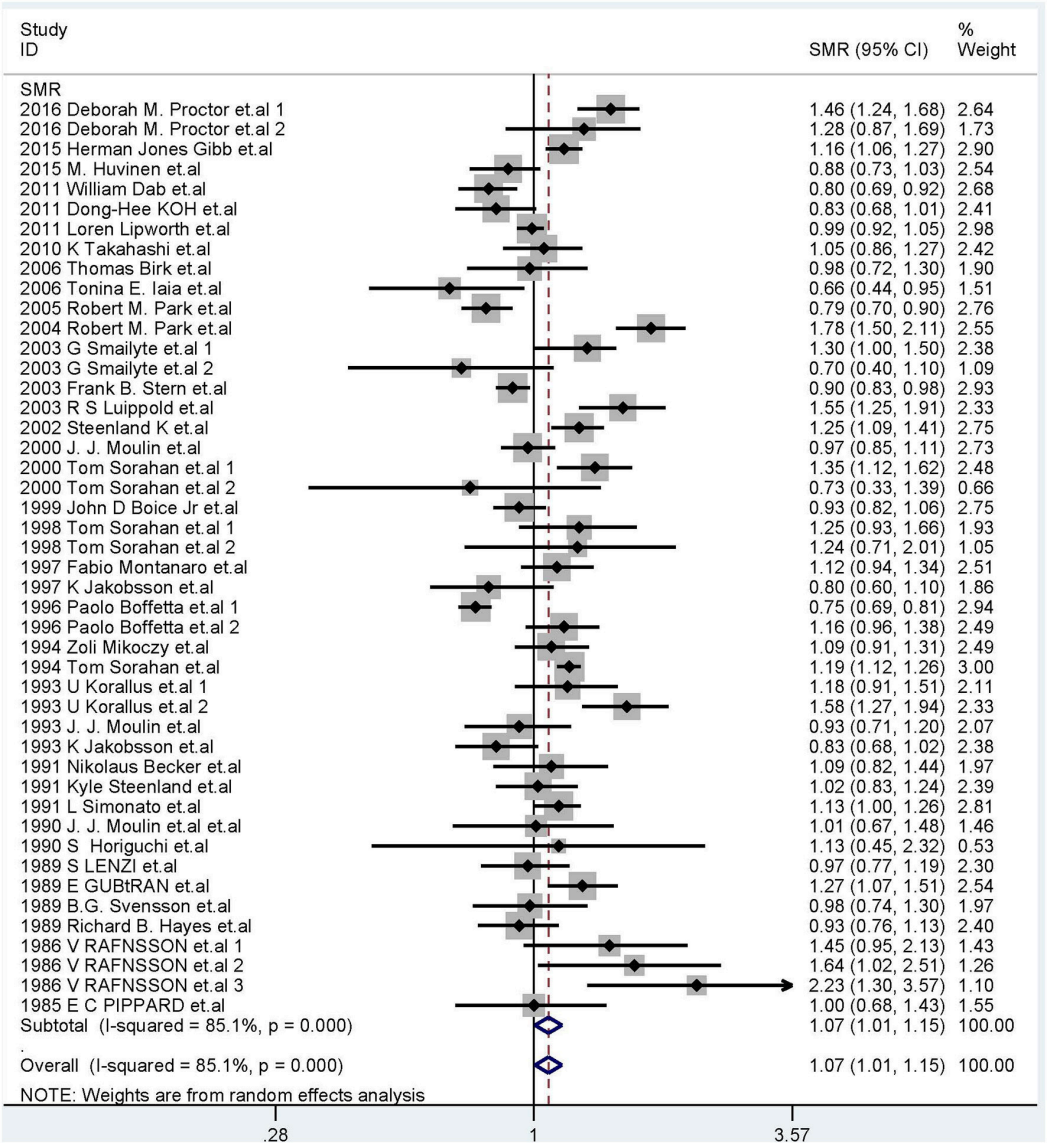
Forest plot of studies included in this meta-analysis of hexavalent chromium with standardized mortality ratios (SMRs) of cancer.

We calculated the overall cancer-specific SMR for all studies and found that significant heterogeneity existed among studies (*I*^2^ = 85.1%, *P* = 0.0001, Figure 2). Therefore, the random-effects model was used and the meta-SMR was found to be 1.07 (95% CI: 1.01–1.15). In subgroup analysis by sex, we found that the SMR was higher for male workers than female ones (SMR = 1.14; 95% CI: 1.06–1.23). When subgroup analysis was conducted by geographical location, we found that Cr(VI) exposure was related to a higher risk of death owing to cancer in North America than in other regions (SMR = 1.19; 95% CI: 1.04–1.35). Among the various occupations, the meta-SMR was higher for chromate production workers (SMR = 1.24; 95% CI: 1.07–1.43) and chromium platers (SMR = 1.22; 95% CI: 1.10–1.34) than for other workers. However, the results differed for workers in Europe and Asia, cement production workers, aircraft manufacturing workers, tanners, welders, and chromium workers.

### Respiratory System Cancers

In total, 44 cohort studies were included, with 94,089 cases; a total of 2,938 patients died of respiratory system cancers. We found that Cr(VI) exposure was correlated with a high-risk of respiratory system cancer mortality (SMR = 1.33; 95% CI: 1.19–1.48). In subgroup analysis, the meta-SMR was higher in male workers than in female workers, and higher in North America and Europe than in Asia. The meta-SMR was elevated for chromate production workers, chromium platers, and welders, but not for cement production workers, aircraft manufacturing workers or tanners.

For lung cancer, the random-effects model was used for the 44 included studies (2,805 deaths), and the meta-SMR was 1.31 (95% CI: 1.17–1.47). In subgroup analysis, the meta-SMR was significantly higher among male workers, in North America, and in Europe than in female workers and in Asia. The meta-SMRs were higher in chromate production workers, chromium platers, and welders than in cement production workers, aircraft manufacturing workers, and tanners.

For larynx cancer, the fixed-effects model was applied for 18 SMR studies (100 deaths) and the meta-SMR was 1.22 (95% CI: 0.98–1.51). The results were robust when we stratified by geographical location; however, in subgroup analysis by occupation, the meta-SMR was higher in chromate production workers than in other workers. In addition, we found that the summary SMR was increased in male workers.

### Digestive System Cancers

The fixed-effects model was used (*I*^2^ = 14.8%, *P* = 0.115) for 99 studies (154,688 cases, 1,833 deaths). There were no significant association between Cr(VI) and death risk of digestive system cancers, and the meta-SMR was 0.97(95% CI: 0.92–1.01). In subgroup analysis, the results were robust for esophageal cancer, stomach cancer, pancreatic cancer, hepatobiliary system cancer, intestinal cancer, colon cancer, and rectal cancer, except for rectal cancer in Europe (Supplementary Table 1).

### Urinary System Cancers

The fixed-effects model was used (*I*^2^ = 35%, *P* = 0.022) for 36 studies (92,532 cases, 170 deaths), and the meta-SMR was 1.20 (95% CI: 1.07–1.35). The meta-SMR was higher in male workers and higher in European workers than in North American workers. The meta-SMR was elevated for welders, but not for chromate production workers, cement production workers, aircraft manufacturing workers or tanners.

For bladder cancer, the fixed-effects model (*I*^2^ = 35.9%, *P* = 0.076) was used for 16 studies (71,950 cases, 157 deaths), and the meta-SMR was 1.24 (95% CI: 1.05–1.47). The meta-SMR was higher in male workers, and higher in European workers than in North American workers, and higher in welders than in tanners.

For kidney cancer, the fixed-effects model (*I*^2^ = 6.10%, *P* = 0.386) was used for 12 studies (62,568 cases, 87 deaths) and the meta-SMR was 1.15 (95% CI: 0.91–1.45). The results were robust when we stratified these studies by geographical location or profession (Supplementary Table 1). The meta-SMR was more significant in male workers than in female ones.

### Lymphatic and Hematopoietic Cancers

The fixed-effects model (*I*^2^ = 10.20%, *P* = 0.276) was used for 47 studies (149,026 cases and 274 deaths), and the meta-SMR was 1.03 (95% CI: 0.93–1.13). The results were robust when we stratified studies by occupation or sex (Supplementary Table 1). In subgroup analysis, exposed workers in Asia had highest cancer mortality. For multiple myeloma (7 studies, 34,112 cases, 46 deaths), the meta-SMR was 1.10 (95% CI: 0.80–1.50). For leukemia (16 studies, 108,957 cases, 186 deaths), the meta-SMR was 1.00 (95% CI: 0.86–1.16). The results were robust in subgroup analysis (Supplementary Table 1). With respect to lymphoma (19 studies, 65,956 cases, 129 deaths), meta-SMR was 1.15 (95% CI: 0.96–1.39). In addition, the meta-SMR was 1.40 (95% CI: 0.87–2.25) for Hodgkin lymphoma and was 1.07 (95% CI: 0.84–1.37) for non-Hodgkin lymphoma.

### Genitourinary System Cancers

We analyzed a total of 27 studies (65,784 cases, 315 deaths) of genitourinary system cancers, and the meta-SMR was 1.04 (95% CI: 0.93–1.17). The meta-SMR for 14 studies (61,178 cases, 254 deaths) of prostate cancer was 0.99 (95% CI: 0.87–1.12). These results were robust in subgroup analysis (Supplementary Table 1). For breast cancer (4 studies, 20,424 cases, 31 deaths), the meta-SMR was 1.12 (95% CI: 0.76–1.65). The fixed-effects model was used (*I*^2^ = 16.90%, *P* = 0.307) for testicular cancer (4 studies, 23,446 cases, 13 deaths), and the meta-SMR was 2.55 (95% CI: 1.38–4.71).

### Other Types of Cancers

For cancers of the buccal cavity and pharynx (17 studies, 58,204 cases, and 126 deaths), the meta-SMR was 0.91 (95% CI: 0.75–1.10). The results were robust in subgroup analysis (Supplementary Table 1). The combined SMR for skin cancer (6 studies, 30,243 cases, 29 deaths) was 0.99 (95% CI: 0.66–1.48). For melanoma, the meta-SMR was 0.90 (95% CI: 0.52–1.54). For connective and soft tissue cancer (3 studies including 20,444 cases and 11 deaths), the combined SMR was 1.22 (95% CI: 0.62–2.41). The meta-SMR for cancers of other sites (23 studies, 53,010 cases, 427 death) was 1.22 (95% CI: 0.98–1.51).

For cancers of the central nervous system, 9 studies (33,718 cases and 50 deaths) were analyzed and the meta-SMR was 1.22 (95% CI: 0.67–2.23). In subgroup analysis, the meta-SMR was highest among Asian workers. For brain cancer, the meta-SMR was 1.67 (95% CI: 0.62–4.46).

For bone cancer (5 studies, 24,568 cases, 12 deaths), the fixed-effects model was used (*I*^2^ = 0%, *P* = 0.422) and the meta-SMR was 2.06 (95% CI: 1.12–3.81). For thyroid cancer (3 studies, 19,109 cases, 8 deaths), the fixed-effects model was used (*I*^2^ = 31.20%, *P* = 0.234) and the combined SMR was 2.41 (95% CI: 1.19–4.87).

### Cancer Incidence

In all, 973,697 workers were involved in 17 SIR studies, and 11,564 of these had cancer. In these studies, the aggregate data covered seven countries and four kinds of occupations. All studies had follow-up periods of more than 15 years; and the detailed data are: follow-up of 15–25 years (9 studies, 52.90%), 26–35 years (4 studies, 23.50%), and 36–45 years (4 studies, 23.50%).

**Figure 3:**
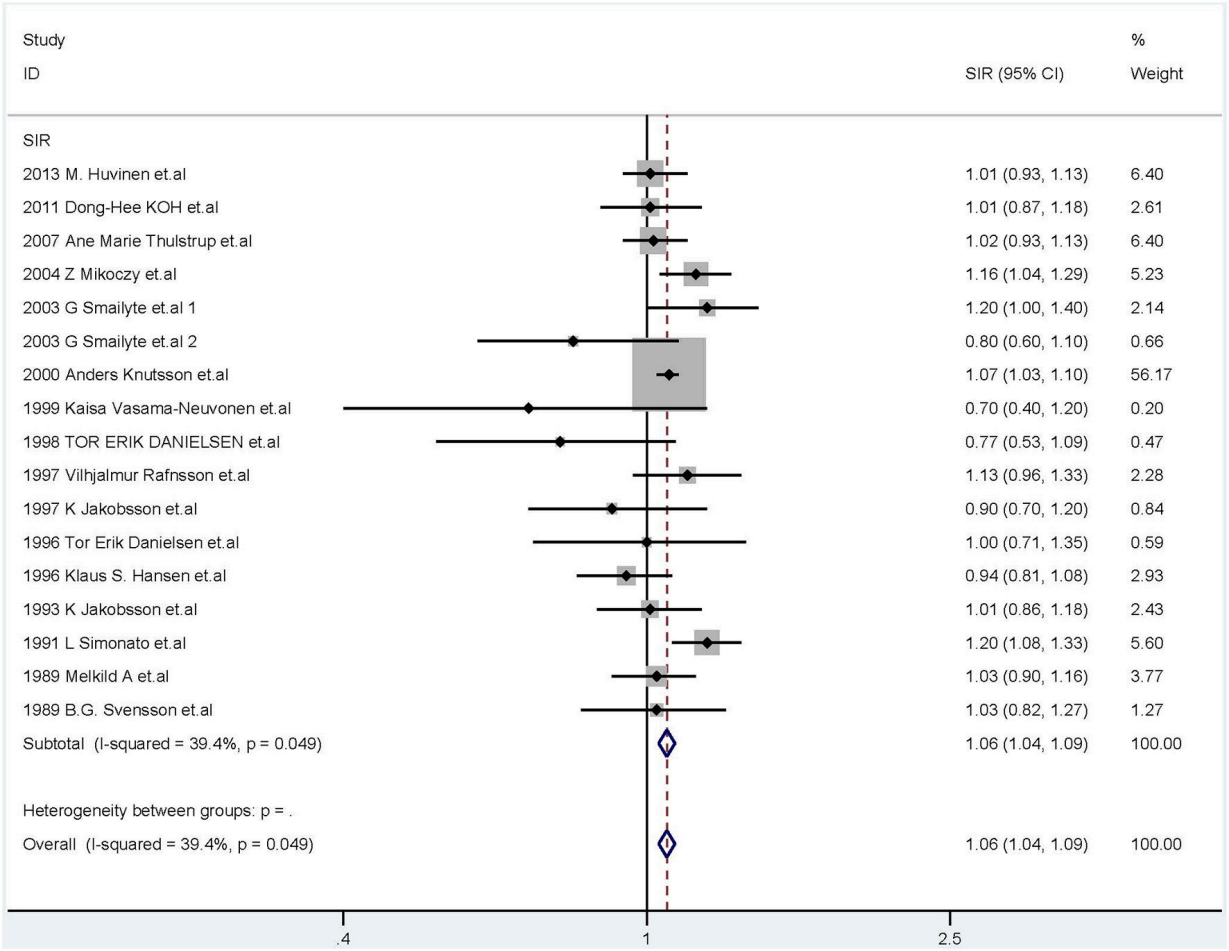
Forest plot of studies included in this meta-analysis of hexavalent chromium with standardized incidence ratios (SIRs) of cancer.

The fixed-effects model was used (*I*^2^ = 39.4%, *P* = 0.049, Figure 3) and the combined SIR was 1.06 (95% CI: 1.04–1.09). In subgroup analysis, the summary SIR was elevated for cement industry workers, and tanners, but not for welders or chromium workers. In addition, workers exposed to Cr(VI) had high cancer incidence in Europe. For workers in North America and Asia, the summary SIRs were above 1.00, but the 95% CIs included 1.00.

### Respiratory System Cancers

The fixed-effects model (*I*^2^ = 5.8%, *P* = 0.375) was used for 30 studies (22,623 cases, 1,001 cancer patients), and the meta-SIR was 1.27 (95% CI: 1.19–1.36). The meta-SIR was higher in male workers and European workers. The meta-SIR was also elevated for welders and cement production workers.

For larynx cancer (10 studies, 61,940 cases, 73 incidents), the meta-SIR was 1.14 (95% CI: 0.89–1.45). A total of 10 studies (69,883 cases, 894 incidents) of lung cancer were analyzed, and the meta-SIR was 1.28 (95% CI: 1.20–1.37) using the fixed-effects model (*I*^2^ = 35.2%, *P* = 0.093). The results were robust in subgroup analysis (Supplementary Table 2). For nasal cancer (3 studies, 10,956 cases, 6 incidents), the meta-SIR was 2.14 (95% CI: 0.79–5.80). The fixed-effects model was used (*I*^2^ = 0%, *P* = 0.764) for 3 studies (34,892 cases, 21 incidents) of pleural mesothelioma, and the meta-SIR was 1.73 (95% CI: 1.08–2.77).

### Digestive System Cancers

The fixed-effects model was used (*I*^2^ = 19.3%, *P* = 0.12) for 51 studies (65,737 cases, 1,407 incidents) of digestive system cancer, and the meta-SIR was 1.05 (95% CI: 1.00–1.11). In subgroup analysis, there were no association between Cr(VI) and esophageal cancer (55,927 cases, 58 incidents), pancreatic cancer (8 studies, 55,248 cases, 160 incidents), colon cancer (19,899 cases, 114 incidents), rectum cancer (66,508 cases, 291 incidents), and hepatobiliary system cancer (55,391 cases, 115 incidents). All these results were robust in subgroup analysis (Supplementary Table 2).

The fixed-effects model was used (*I*^2^ = 43.30%, *P* = 0.042) for 14 studies (66,508 cases, 420 incidents) of stomach cancer, and the meta-SIR was 1.20 (95% CI: 1.08–1.32). In subgroup analysis, meta-SIR was elevated in male workers, European workers, and cement production workers, but not in welders. For the 14 studies (66,508 cases, 668 incidents) of bowel cancer (intestine, colon, and rectum), the meta-SIR was 1.03 (95% CI: 0.96–1.12), with the SIR being especially high in welders.

### Urinary System Cancers

The fixed-effects model was used (*I*^2^ = 0%, *P* = 0.6) for 23 studies (62,986 cases, 562 incidents) of urinary system cancer, and the meta-SIR was 1.03 (95% CI: 0.95–1.13). In 9 studies of bladder cancer (60,489 cases, 360 incidents), the meta-SIR was 1.09 (95% CI: 0.98–1.22). In the 10 studies (54,757 cases, 175 incidents) of kidney cancer analyzed, the meta-SIR was 0.93 (95% CI: 0.79–1.08). These results were robust in subgroup analysis (Supplementary Table 2).

### Genitourinary System Cancers

For the 13 studies (61,564 cases, 1,083 incidents) of male genital cancer and 13 studies (61,564 cases, 1,047 incidents) of prostate cancer, the combined SIR was 1.14 (95% CI: 1.07–1.21) and 1.15 (95% CI: 1.08–1.22), respectively, with the SIR being especially high among European workers. These results were robust when we stratified these studies by occupation (Supplementary Table 2).

For 8 studies (903,535 cases, 5,181 incidents) of female genital cancer, the meta-SIR was 1.02 (95% CI: 0.85–1.23). The results were robust in subgroup analysis (Supplementary Table 2). For breast cancer, the meta-SIR was 1.08 (95% CI: 0.84–1.38).

### Lymphatic and Hematopoietic Cancer

The fixed-effects model was used (*I*^2^ = 0%, *P* = 0.998) for 24 studies (633,367 cases, 432 incidents) of lymphatic and hematopoietic cancers, and the meta-SIR was 1.10 (95% CI: 1.00–1.22). The results were robust in subgroup analysis (Supplementary Table 2). For multiple myeloma (5 studies, 50,444 cases, 72 incidents), the meta-SIR was 1.13 (95% CI: 0.89–1.44). In the 7 studies of leukemia (53,390 cases, 148 incidents) analyzed, the meta-SIR was 1.11 (95% CI: 0.93–1.31). For lymphoma, the combined SIR of 5 studies (88,894 cases, 173 incidents) was 1.12 (95% CI: 0.96–1.30). In addition, the meta-SIR was 0.99 (95% CI: 0.65–1.52) for Hodgkin lymphoma, and 1.14 (95% CI: 0.97–1.34) for non-Hodgkin lymphoma.

### Oral Cancers

The fixed-effects model was used (*I*^2^ = 4.3%, *P* = 0.404) for 16 studies (59,537 cases, 163 incidents) of oral cancer (buccal cavity and pharynx), and the meta-SIR was 1.30 (95% CI: 1.11–1.54), with the SIR being especially high in male workers, Europeans and welders (Supplementary Table 2).

### Cancer of Other Sites

The meta-SIR for 4 studies (48,417 cases, 29 incidents) of thyroid cancer was 0.81 (95% CI: 0.54–1.21). Three studies of brain cancer (42,821 cases, 134 incidents) were analyzed, and the meta-SIR was 1.04 (95% CI: 0.87–1.24). The combined SIR for 13 studies (55,880 cases, 432 incidents) of skin cancer was 1.02 (95% CI: 0.95–1.10). For melanoma, 3 studies (43,802 cases, 117 incidents) were analyzed, and the meta-SIR was 0.92 (95% CI: 0.77–1.11). The meta-SIR for soft tissue cancer (3 studies, 43,676 cases, 35 incidents) was 1.20 (95% CI: 0.84–1.71). The meta-SIR of other cancers (5 studies, 43,511 cases, 180 incidents) was 1.09 (95% CI: 0.94–1.26).

### Exposure Level of Cr(VI) With Incidence and Mortality Risk of Cancers

As shown in Table 3, in the subgroup analysis of cumulative hexavalent exposure (mg Cr(VI)/m^3^-years), the higher the concentration of Cr(VI) in the air, the higher the risk of cancer death for workers. When the concentration is more than 1 mg Cr(VI)/m^3^, there is a significant increase of risk of cancer death. As for time since first exposure to Cr(VI), an elevated death risk of cancer was observed, especially for lung cancer.

**Table 3:** The results of the association between different exposure level of Cr(VI) and incidence and mortality of human cancers.

**Exposure level assessment**	**Number of study**	**Cancer type**	**Effect model**	**Heterogeneity**		**Outcome**	**Effect value**	**LL**	**UL**
				***I*^**2**^ (%)**	***P*-Value**			
**CUMULATIVE HEXAVALENT EXPOSURE (MG CR(VI)/m**^**3**^**-YEARS)**									
<0.5	3	All	Fixed	0.00	0.41	SMR	1.35	0.81	2.25
0.5–1	2	All	Fixed	0.00	0.33	SMR	1.50	0.91	2.48
>1	2	All	Fixed	0.00	0.51	SMR	4.17	2.95	5.90
Total	7	All	Random	71.60	0.01	SMR	2.48	1.93	3.18
**TIME SINCE FIRST EXPOSURE (YEARS)**									
<20	17	All	Random	50.10	0.01	SMR	1.42	1.18	1.71
≥20	19	All	Fixed	10.30	0.33	SMR	1.55	1.27	1.89
Total	36	All	Fixed	36.20	0.02	SMR	1.53	1.38	1.71
<20	14	Lung cancer	Fixed	0.00	0.55	SMR	1.52	1.24	1.85
≥20	15	Lung cancer	Fixed	49.80	0.02	SMR	1.57	1.36	1.81
Total	29	Lung cancer	Fixed	29.50	0.07	SMR	1.55	1.38	1.74
**DURATION OF EMPLOYMENT (YEARS)**									
1–10	20	All	Fixed	47.70	0.02	SMR	1.11	1.05	1.18
11–20	5	All	Fixed	20.10	0.29	SMR	0.98	0.92	1.05
≥21	12	All	Random	90.30	0.01	SMR	0.92	0.87	0.96
Total	37	All	Random	80.3	0.01	SMR	0.99	0.96	1.02
1–10	18	Lung cancer	Fixed	0.01	0.48	SMR	1.32	1.21	1.43
11–20	4	Lung cancer	Fixed	0.01	0.58	SMR	1.09	0.97	1.23
≥21	10	Lung cancer	Random	91.80	0.01	SMR	1.43	1.01	2.02
Total	32	Lung cancer	Random	80.50	0.01	SMR	1.40	1.21	1.63
1–10	2	Colorectal cancer	Fixed	0.01	0.91	SMR	1.08	0.87	1.34
11–20	2	Colorectal cancer	Fixed	49.70	0.16	SMR	1.00	0.79	1.27
≥21	3	Colorectal cancer	Random	56.10	0.10	SMR	1.03	0.89	1.19
Total	7	Colorectal cancer	Fixed	11.70	0.34	SMR	1.04	0.93	1.16
1–14	6	All	Fixed	0.00	0.88	SIR	1.13	0.90	1.40
≥15	4	All	Fixed	0.00	0.30	SIR	1.34	1.13	1.60
Total	10	All	Fixed	0.00	0.64	SIR	1.25	1.09	1.44

AN interesting finding is that a slight increase of death risk of cancer was observed in workers less 10 years, when the subgroup analysis of duration of employment about Cr(VI) was conducted. Further analysis revealed that long duration of exposure to Cr(VI) was associated with elevated death risk for lung cancer, but not for colorectal cancer. In SIR studies, the duration of employment was related to increased cancer risk, especially when workers were employed more than 15 years.

### Publication Bias

**Figure 4:**
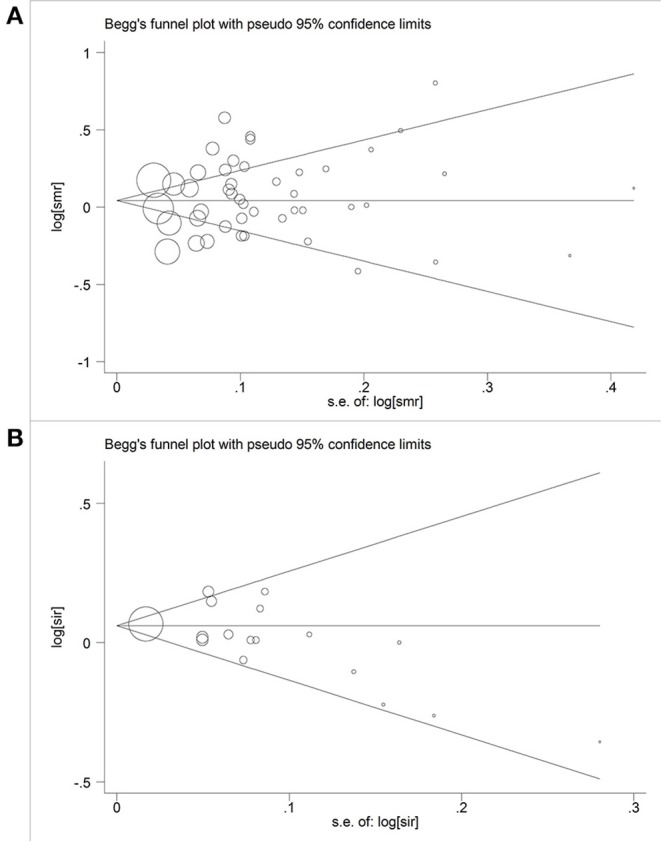
Begger's funnel plot of studies included in this meta-analysis of hexavalent chromium with cancer mortality and morbidity. **(A)** Begger's funnel plot of hexavalent chromium and SMR; **(B)** Begger's funnel plot of hexavalent chromium and SIR.

Begg's funnel plot and Egger's test were used to investigate publication bias. We found no evidence of asymmetry in the funnel plot of all studies combined (Figure 4), or in the funnel plots for each subgroup analysis (Supplementary Table 2). The results of the Egger's (SMR: *P* = 0.36; SIR: *P* = 0.09) and Begg's tests (SMR: *P* = 0.78; SIR: *P* = 0.14) also showed no significant evidence of publication bias.

### Sensitivity Analysis

**Figure 5:**
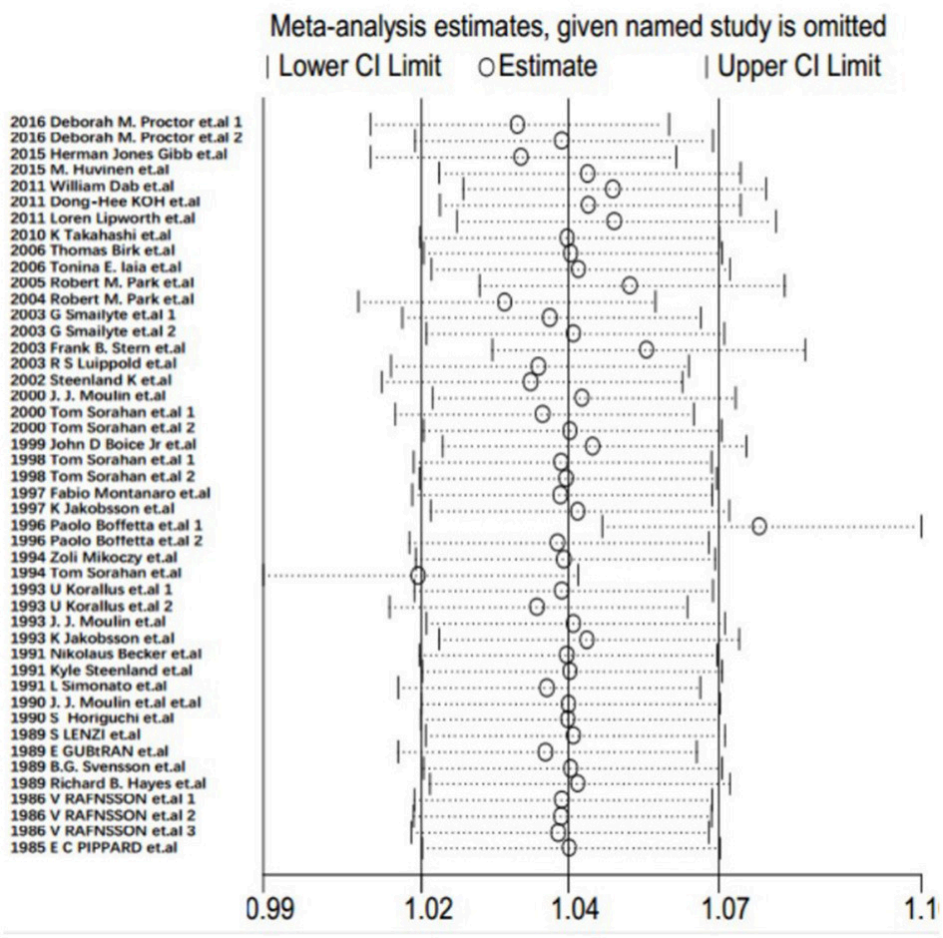
Results of sensitivity analysis for SMRs.

**Figure 6:**
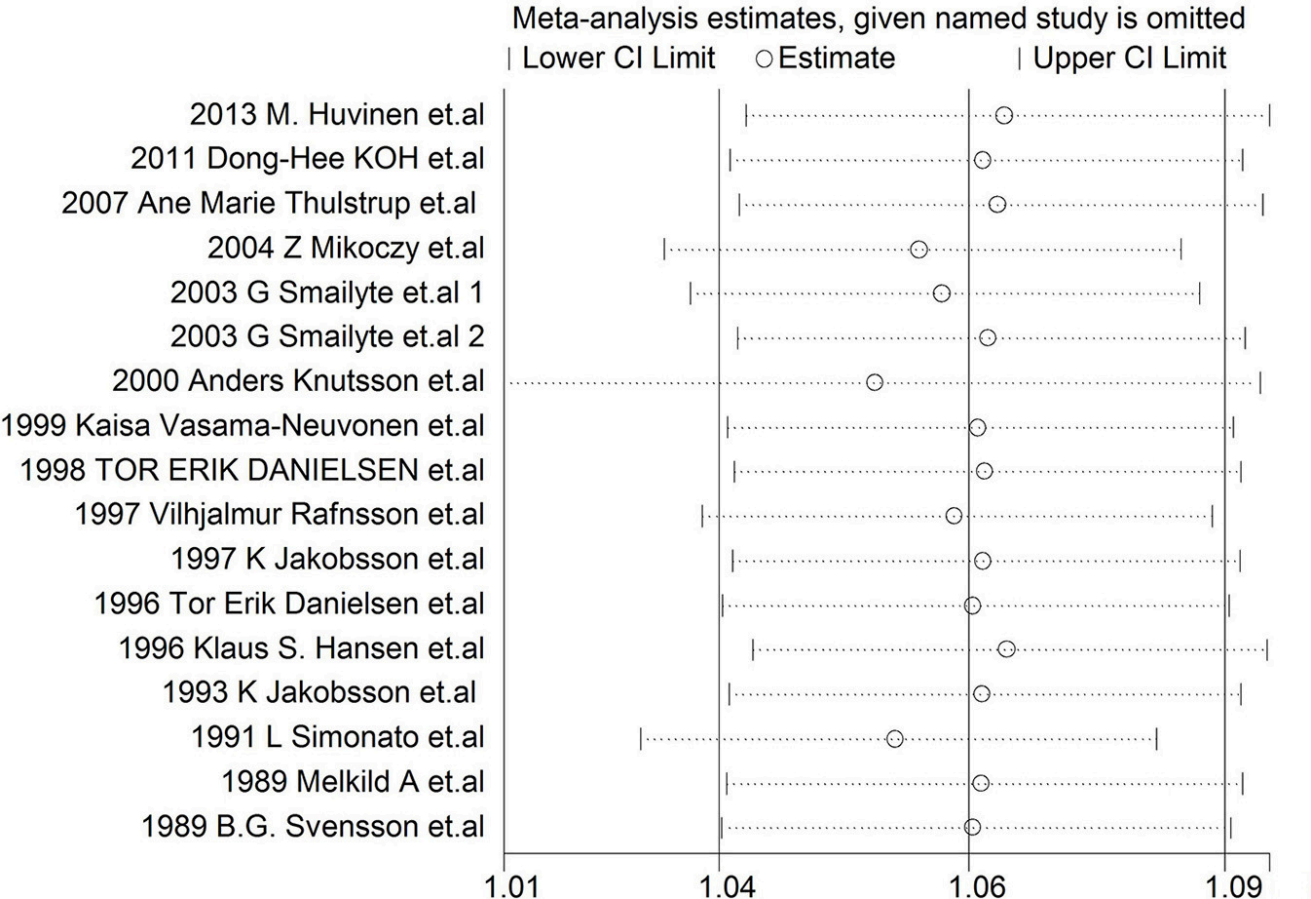
Results of sensitivity analysis for SIRs.

Each study was individually eliminated to assess the effect of individual studies on the results. The sensitive analysis (Figure 5) showed that the results were significantly influenced by two studies (31, 33). Therefore, the meta-SMR was calculated after excluding these two studies, resulting in a slightly higher meta-SMR (1.08 vs. 1.07). As shown in Figure 6, there was no significant change in the merged SIRs, which indicated the stability of results.

## Discussion

### Summary of the Main Findings

In this meta-analysis, we identified 47 studies specifically investigating cancer mortality or incidence in relation to Cr(VI) exposure among workers. Overall, our meta-analysis provided evidence that Cr(VI) might cause cancers of the respiratory system, buccal cavity and pharynx, prostate, and stomach in humans. This findings differ from the conclusions of Donato et al. (57), who suggested that Cr(VI) was related to increased risk of cancer incidence of the lung, larynx, bladder, kidney, testis, thyroid, and bone. In addition, the incidence and death risk of cancer was associated significantly with concentration of Cr(VI) in the air and the exposure time.

The overall cancer mortality risk of 1.07 indicated that workers exposed to Cr(VI) had a higher risk of cancer death compared with their age-matched and sex-matched counterparts in the general population. For lung cancer, a significant relationship existed between exposure to Cr(VI) and cancer death. This result was consistent with a previous meta-analysis conducted by Cole et al. that included 49 SMR epidemiological studies and showed that Cr(VI) was a weak cause of lung cancer (58). Moreover, we found that Cr(VI) was related to a higher risk of larynx cancer mortality, especially among chromate production workers. The meta-analysis did not find evidence for increased risk of death due to digestive system cancers, which was accordant with a formal meta-analysis performed by Proctor et al. Those authors analyzed 32 SMR studies and found that workers exposed to Cr(VI) were not at a higher risk of death owing to gastrointestinal tract cancer (59). However, our analysis of 14 studies showed that the meta-SMR was high for rectal cancer in Europe; these results must be confirmed in further studies.

A major finding here was that for workers exposed to Cr(VI), the mortality risk of urinary system cancer and bladder cancer was high. Among male workers, the meta-SMR of kidney cancer was high. In addition, the present meta-analysis provided additional evidence for elevated risk of death owing to testicular, bone, and thyroid cancers among workers exposed to Cr(VI), however, additional epidemiological evidence is needed to verify the accuracy of these results. However, the associations between exposure to Cr(VI) and the high mortality of some cancers were not significant, like those of breast, lymphatic, and hematopoietic, buccal cavity and pharynx, central nervous system, skin, and connective and soft tissue cancers.

The summary SIR of 1.06 (95% CI: 1.04–1.09) provided evidence for increased risk of cancer among workers exposed to Cr(VI), as compared with the general population. The narrow CI, which excludes 1.0, and the low *I*^2^ value indicate that this result was not due to chance, to a large extent. We also performed subgroup analysis by sex, occupation, cancer type, and geographical location to explore more specific relationships. The results were robust for cement industry workers and tanners, especially in Europe. For studies in North America and Asia, the summary SIR was above 1.00, but the 95% CI included 1.00. For respiratory system cancers, the meta-SIR was elevated, especially in male workers, European workers and welders. The associations were strong and robust for lung cancer and pleural mesothelioma, but not for larynx cancer and nasal cancers. Kim et al. suggested that there was no strong evidence of an association of Cr(VI) with nasal and paranasal cancers (60). However, in 2015, Binazzi et al. conducted a meta-analysis of risk ratios in 28 studies (11 cohort, 17 case-control) and suggested that sinonasal cancer was associated with exposure to nickel and chromium compounds (61). Therefore, more studies of these cancer types are urgently needed.

A novel finding was that workers exposed to Cr(VI) were at risk of oral cancer. Yuan et al. suggested that nickel and chromium play a role in oral cancer (62). The present meta-analysis showed that the risk for genital cancers, such as prostate cancer, was elevated in male workers. With respect to the digestive system, the summary SIR was elevated among exposed workers only for stomach cancer and, not for esophageal, pancreatic, intestinal, colon, rectal, or hepatobiliary system cancers. This finding is in agreement with the results of the meta-analysis performed by Welling et al. (4), as previously described. These results were also consistent with the conclusion of a previous meta-analysis of pancreatic cancer by Ojajarvi et al. Those authors analyzed 92 studies on occupational exposures and found that the meta-risk ratio for chromium was 1.40 (95% CI: 0.90–2.30) (63); the established causal relationship between exposure to Cr(VI) and lymphatic and hematopoietic cancer was found to be weak.

The cohort studies included here were carried out during different time periods, which may account in part for the heterogeneity, even though all follow up periods of the included studies were more than 5 years. However, the distribution of the six follow-up periods in the included SMR studies was well-proportioned, so the heterogeneity may be alleviated to a certain extent. The proportion of occupation types included in the analysis varied: chromate production workers, welders, and tanners comprised nearly 20% each, whereas, other occupations comprised 5–10%. The varying exposure times may be another source of heterogeneity, even though the exposure times of the included cohorts were mostly over 1 year. To some extent, the study populations included in the meta-analysis, which were from different geographical regions, may cause bias and heterogeneity. The SMR is a reflection of relative ratio and depends on the adjustment of confounders in the study and reference populations. Most studies in this meta-analysis included adjustment for confounding factors, such as age and sex.

There is some evidence that clarifies the carcinogenesis of Cr(VI) exposure. In 2014, Ovesen declared that long-term exposure to low-concentrations of Cr(VI) could induce DNA damage (64). Wang et al. found that chronic Cr(VI) exposure is associated with epigenetic dysregulation via an increase in the related histone-lysing methyltransferases expression which plays an essential role in Cr(VI)-induced cancer stem cell-like property and cell transformation (65). Another study confirmed that Cr(VI) can form protein-Cr-DNA adducts and silence tumor suppressor genes, as well as disrupt CTCF binding and nucleosome spacing (66). In addition, Clementino stated that Cr(VI)is related to oxidative stress and metabolic reprogramming, which contribute to tumorigenesis by participating in enhancement of the anti-apoptosis ability and rapid proliferation of cells (67). Therefore, additional high-qualify studies are needed to further explore the carcinogenesis of Cr(VI) exposure.

### Limitations

There are some limitations in the present meta-analysis. Although we used the Begg's funnel plot and Egger's test and found no publication bias, slight publication bias is unavoidable. Owing to the occupational specificity and sex limitations, the study population comprised mostly male workers and specific sex-related SMRs were lacking. Therefore, the finding that high cancer deaths existed among male workers is acceptable. In addition, in a cohort study from 1971 to 1986 among 33,503 concrete workers, Knutsson et al. found that risk of cancer was high in female concrete workers (SIR = 1.17; 95% CI: 1.03–1.10) (50); further evidence is needed to verify this relationship. It should be noted that all studies included in this analysis comprised adults rather than children as the study population, as well as workers exposed to Cr(VI) rather than the general population; therefore, the results should not be generalized or applied to populations other than those who are exposed to Cr(VI). An additional limitation was that the included cohorts were from Europe, North America and Asia, with no available reports from other geographical areas available. For SMR studies, 70% were from Europe, 22% from North America, and 8% from Asia. For SIR studies, 82% were from Europe, 12% from North America, and 6% from Asia. Therefore, the estimates are largely dominated by the European cohorts. Nevertheless, these cohorts may provide more accurate and consistent baseline data compared with others owing to their large sample size and similar geographical conditions.

## Conclusions

In summary, our meta-analysis provides evidence to support the association between exposure to Cr(VI) and increased mortality and incidence of some cancers. Based on our results, Cr(VI) exposure is related to a high-risk of death owing to lung, larynx, bladder, kidney, testicular, bone, and thyroid cancer. In addition, Cr(VI) exposed workers are at elevated risk of cancers of the respiratory system, buccal cavity, pharynx, prostate, and stomach. As with all meta-analyses, publication bias, and heterogeneity cannot be entirely eliminated. These findings require the support of well-designed cohort studies which are capable of addressing the problem of accurate measurement of exposure dose and time and potential confounders in the relationship between exposure to Cr(VI) and cancer.

## Author Contributions

All authors read, critically reviewed, and approved the final manuscript. YD and MW conducted the database searches, screened titles, abstracts and full-texts for eligibility, performed study quality assessments. ZD and GZ planned and designed the research. CD and PX provided methodological support, advice. SL tested the feasibility of the study. QH, ZZ, YZ, YW, and QH extract data. LZ performed the statistical analysis. YD and TT wrote the manuscript.

### Conflict of Interest Statement

The authors declare that the research was conducted in the absence of any commercial or financial relationships that could be construed as a potential conflict of interest.

## Abbreviations

SMR: standardized mortality ratio
SIR: standardized incidence ratio
CI: confidence interval
LL: lower limit
UL: upper limit
NOS: The Newcastle-Ottawa scale
Cr(VI): hexavalent chromium.
