# Supplementary material for: The Effect of Hexavalent Chromium on the Incidence and Mortality of Human Cancers: A Meta-Analysis Based on Published Epidemiological Cohort Studies
Source: Front Oncol. 2019 Feb 4;9:24. doi: 10.3389/fonc.2019.00024 (PMC6369173; doi:10.3389/fonc.2019.00024)
Supplement: Supplementary file 1 [file Table_1.DOCX]

**The effect of hexavalent chromium on the incidence and mortality of human cancers: A meta-analysis based on published epidemiological cohort studies**

**Authors:** Yujiao Deng1,2,†, Meng Wang1,2,†, Tian Tian1,2,†, Shuai Lin1, Peng Xu1, Linghui Zhou1, Cong Dai1, Qian Hao1, Ying Wu1, Zhen Zhai1, Yue Zhu1, Guihua Zhuang3, and Zhijun Dai1,2*

***Correspondence to:** Zhijun Dai, (E-Mail: dzj0911@126.com), or Guihua Zhuang, (E-Mail: [zhuanggh@xjtu.edu.cn](mailto:zhuanggh@xjtu.edu.cn)).

**Supplementary table 1. The results of all subgroup analysis for standardized mortality ratio (SMR)**

| Cancer Type | No of study | Sex | District | Profession | Model Type | I^2^ (%) | P value | Egger test (p VALUE) | SMR(95%CIs) |  | | |
| --- | --- | --- | --- | --- | --- | --- | --- | --- | --- | --- | --- | --- |
| All types of cancer | 43 | mix | All | All | random | 85.10 | 0.00 | 0.36 | 1.07(1.01,1.15) |  | | |
| All types of cancer | 10 | mix | North America | All | random | 90.80 | 0.00 |  | 1.19(1.04,1.35) |  | | |
| All types of cancer | 29 | mix | Europe | All | random | 82.30 | 0.08 |  | 1.06(0.97,1.15) |  | | |
| All types of cancer | 4 | mix | Asia | All | random | 52.50 | 0.10 |  | 0.88(0.75,1.03) |  | | |
| All types of cancer | 13 | mix | All | All | random | 84.20 | 0.00 |  | 0.97(0.88,1.07) |  | | |
| All types of cancer | 27 | male | All | All | random | 79.00 | 0.00 |  | 1.14(1.06,1.23) |  | | |
| All types of cancer | 3 | female | All | All | fixed | 26.30 | 0.26 |  | 0.88(0.60,1.29) |  | | |
| All types of cancer | 8 | mix | All | Chromate production workers | random | 79.00 | 0.00 |  | 1.24(1.07,1.43) |  | | |
| All types of cancer | 5 | mix | All | Cement production workers | random | 76.90 | 0.00 |  | 0.89(0.73,1.09) |  | | |
| All types of cancer | 2 | mix | All | Aircraft manufacturing workers | fixed | 0.00 | 0.40 |  | 0.98(0.92,1.04) |  | | |
| All types of cancer | 7 | mix | All | Chromium platers | fixed | 0.00 | 0.46 |  | 1.22(1.10,1.34) |  | | |
| All types of cancer | 8 | mix | All | Tanners | random | 82.60 | 0.00 |  | 0.95(0.83,1.09) |  | | |
| All types of cancer | 11 | mix | All | Welders | random | 79.10 | 0.00 |  | 1.02(0.92,1.13) |  | | |
| All types of cancer | 2 | mix | All | Chromium workers | random | 95.80 | 0.00 |  | 1.29(0.68,2.44) |  | | |
| All types of cancer | 3 | mix | All | Masons | fixed | 0.00 | 0.42 |  | 1.69(1.31,2.19) |  | | |
| Respiratory system cancer | 66 | mix | All | All | random | 81.70 | 0.00 | 0.56 | 1.33(1.19,1.48) |  |  |  |
| Respiratory system cancer | 14 | mix | North America | All | random | 87.40 | 0.00 |  | 1.34(1.11,1.62) |  |  |  |
| Respiratory system cancer | 48 | mix | Europe | All | random | 80.80 | 0.00 |  | 1.36(1.17,1.57) |  |  |  |
| Respiratory system cancer | 4 | mix | Asia | All | fixed | 37.80 | 0.19 |  | 1.06(0.77,1.45) |  |  |  |
| Respiratory system cancer | 41 | male | All | All | random | 54.90 | 0.00 |  | 1.48(1.35,1.62) |  |  |  |
| Respiratory system cancer | 22 | mix | All | All | random | 84.80 | 0.00 |  | 1.11(0.91,1.35) |  |  |  |
| Respiratory system cancer | 3 | female | All | All | fixed | 0.00 | 0.94 |  | 1.23(0.77,1.97) |  |  |  |
| Respiratory system cancer | 11 | mix | All | Chromate production workers | random | 67.50 | 0.00 |  | 1.68(1.42,1.99) |  |  |  |
| Respiratory system cancer | 6 | mix | All | Cement production workers | fixed | 47.10 | 0.09 |  | 1.02(0.85,1.21) |  |  |  |
| Respiratory system cancer | 4 | mix | All | Aircraft manufacturing workers | fixed | 0.00 | 0.94 |  | 0.97(0.87,1.07) |  |  |  |
| Respiratory system cancer | 10 | mix | All | Chromium platers | fixed | 0.00 | 0.53 |  | 1.43(1.23,1.65) |  |  |  |
| Respiratory system cancer | 12 | mix | All | Tanners | random | 87.30 | 0.00 |  | 1.12(0.80,1.57) |  |  |  |
| Respiratory system cancer | 18 | mix | All | Welders | fixed | 0.70 | 0.45 |  | 1.39(1.30,1.47) |  |  |  |
| Lung cancer | 44 | mix | All | All | random | 86.20 | 0.00 | 0.70 | 1.31(1.17,1.47) |  |  |  |
| Lung cancer | 10 | mix | North America | All | random | 91.30 | 0.00 |  | 1.38(1.12,1.69) |  |  |  |
| Lung cancer | 30 | mix | Europe | All | random | 85.60 | 0.00 |  | 1.31(1.12,1.54) |  |  |  |
| Lung cancer | 4 | mix | Asia | All | fixed | 33.70 | 0.21 |  | 1.09(0.80,1.47) |  |  |  |
| Lung cancer | 29 | male | All | All | random | 66.50 | 0.00 |  | 1.49(1.35,1.65) |  |  |  |
| Lung cancer | 12 | mix | All | All | random | 87.00 | 0.00 |  | 0.99(0.81,1.21) |  |  |  |
| Lung cancer | 3 | female | All | All | fixed | 0.00 | 0.94 |  | 1.23(0.77,1.97) |  |  |  |
| Lung cancer | 9 | mix | All | Chromate production workers | random | 73.80 | 0.00 |  | 1.69(1.42,2.01) |  |  |  |
| Lung cancer | 5 | mix | All | Cement production workers | random | 51.40 | 0.08 |  | 1.01(0.76,1.36) |  |  |  |
| Lung cancer | 7 | mix | All | Chromium platers | fixed | 10.40 | 0.35 |  | 1.42(1.23,1.64) |  |  |  |
| Lung cancer | 7 | mix | All | Tanners | random | 81.60 | 0.00 |  | 0.90(0.68,1.18) |  |  |  |
| Lung cancer | 10 | mix | All | Welders | fixed | 42.90 | 0.07 |  | 1.38(1.30,1.47) |  |  |  |
| Larynx cancer | 18 | mix | All | All | fixed | 0.00 | 0.94 | 0.35 | 1.22(0.98,1.51) |  |  |  |
| Larynx cancer | 6 | mix | North America | All | fixed | 0.00 | 0.79 |  | 1.26(0.89,1.80) |  |  |  |
| Larynx cancer | 12 | mix | Europe | All | fixed | 0.00 | 0.84 |  | 1.19(0.90,1.56) |  |  |  |
| Larynx cancer | 10 | male | All | All | fixed | 0.00 | 0.98 |  | 1.43(1.03,1.99) |  |  |  |
| Larynx cancer | 8 | mix | All | All | fixed | 0.00 | 0.67 |  | 1.08(0.81,1.44) |  |  |  |
| Larynx cancer | 3 | mix | All | Chromate production workers | fixed | 0.00 | 0.94 |  | 1.92(1.03,3.56) |  |  |  |
| Larynx cancer | 4 | mix | All | Tanners | fixed | 0.00 | 0.61 |  | 1.00(0.62,1.61) |  | |  |
| Larynx cancer | 6 | mix | All | Welders | fixed | 0.00 | 0.94 |  | 1.36(0.99,1.87) |  |  |  |
| Digestive system cancer | 99 | mix | All | All | fixed | 14.80 | 0.12 | 0.45 | 0.97(0.92,1.01) |  |  |  |
| Digestive system cancer | 17 | mix | North America | All | fixed | 0.00 | 0.91 |  | 0.99(0.91,1.07) |  |  |  |
| Digestive system cancer | 73 | mix | Europe | All | fixed | 21.00 | 0.06 |  | 0.99(0.93,1.06) |  |  |  |
| Digestive system cancer | 9 | mix | Asia | All | fixed | 0.00 | 0.48 |  | 0.82(0.72,0.93) |  |  |  |
| Digestive system cancer | 57 | male | All | All | fixed | 20.30 | 0.10 |  | 0.97(0.91,1.04) |  |  |  |
| Digestive system cancer | 40 | mix | All | All | fixed | 10.60 | 0.28 |  | 0.96(0.90,1.03) |  |  |  |
| Digestive system cancer | 2 | female | All | All | fixed | 0.00 | 0.37 |  | 0.73(0.28,1.89) |  |  |  |
| Digestive system cancer | 10 | mix | All | Chromate production workers | fixed | 12.70 | 0.33 |  | 0.90(0.75,1.10) |  |  |  |
| Digestive system cancer | 12 | mix | All | Cement production workers | fixed | 0.00 | 0.96 |  | 0.84(0.72,1.00) |  |  |  |
| Digestive system cancer | 12 | mix | All | Aircraft manufacturing workers | fixed | 0.00 | 0.79 |  | 1.00(0.89,1.13) |  | |  |
| Digestive system cancer | 9 | mix | All | Chromium platers | fixed | 16.00 | 0.30 |  | 1.12(0.85,1.47) |  |  |  |
| Digestive system cancer | 20 | mix | All | Tanners | fixed | 41.10 | 0.03 |  | 0.97(0.83,1.12) |  |  |  |
| Digestive system cancer | 23 | mix | All | Welders | fixed | 32.40 | 0.07 |  | 0.95(0.85,1.08) |  |  |  |
| Stomach cancer | 33 | mix | All | All | random | 53.00 | 0.00 | 0.27 | 0.93(0.78,1.09) |  |  |  |
| Stomach cancer | 4 | mix | North America | All | random | 0.00 | 0.40 |  | 0.77(0.56,1.04) |  |  |  |
| Stomach cancer | 25 | mix | Europe | All | random | 54.10 | 0.00 |  | 1.00(0.81,1.23) |  | |  |
| Stomach cancer | 4 | mix | Asia | All | random | 0.00 | 0.78 |  | 0.73(0.58,1.00) |  |  |  |
| Stomach cancer | 23 | male | All | All | random | 51.10 | 0.00 |  | 0.96(0.78,1.18) |  |  |  |
| Stomach cancer | 9 | mix | All | All | random | 58.20 | 0.01 |  | 0.84(0.62,1.16) |  |  |  |
| Stomach cancer | 6 | mix | All | Chromate production workers | random | 51.90 | 0.07 |  | 0.87(0.50,1.54) |  |  |  |
| Stomach cancer | 5 | mix | All | Cement production workers | fixed | 0.00 | 0.83 |  | 0.78(0.58,1.06) |  |  |  |
| Stomach cancer | 2 | mix | All | Aircraft manufacturing workers | fixed | 0.00 | 0.35 |  | 0.80(0.57,1.12) |  |  |  |
| Stomach cancer | 3 | mix | All | Chromium platers | random | 60.80 | 0.08 |  | 1.07(0.54,2.11) |  |  |  |
| Stomach cancer | 6 | mix | All | Tanners | random | 73.20 | 0.00 |  | 0.81(0.48,1.36) |  |  |  |
| Stomach cancer | 7 | mix | All | Welders | random | 71.70 | 0.00 |  | 1.05(0.73,1.51) |  |  |  |
| Esophagus cancer | 14 | mix | All | All | fixed | 0.00 | 0.96 | 0.57 | 0.88(0.73,1.05) |  |  |  |
| Esophagus cancer | 4 | mix | North America | All | fixed | 0.00 | 0.62 |  | 1.02(0.78,1.35) |  |  |  |
| Esophagus cancer | 9 | mix | Europe | All | fixed | 0.00 | 0.99 |  | 0.78(0.62,1.00) |  | |  |
| Esophagus cancer | 7 | male | All | All | fixed | 0.00 | 0.67 |  | 0.86(0.65,1.14) |  |  |  |
| Esophagus cancer | 7 | mix | All | All | fixed | 0.00 | 0.95 |  | 0.89(0.71,1.12) |  |  |  |
| Esophagus cancer | 2 | mix | All | Cement production workers | fixed | 0.00 | 1.00 |  | 0.76(0.42,1.37) |  |  |  |
| Esophagus cancer | 2 | mix | All | Aircraft manufacturing workers | fixed | 0.00 | 0.82 |  | 0.96(0.68,1.36) |  |  |  |
| Esophagus cancer | 6 | mix | All | Tanners | fixed | 0.00 | 0.87 |  | 0.76(0.55,1.03) |  |  |  |
| Esophagus cancer | 2 | mix | All | Welders | fixed | 0.00 | 0.98 |  | 0.83(0.53,1.29) |  |  |  |
| Hepatobiliary system cancer | 16 | mix | All | All | fixed | 4.40 | 0.40 | 0.30 | 0.91(0.79,1.04) |  |  |  |
| Hepatobiliary system cancer | 3 | mix | North America | All | fixed | 0.00 | 0.69 |  | 0.98(0.72,1.34) |  |  |  |
| Hepatobiliary system cancer | 9 | mix | Europe | All | fixed | 29.70 | 0.18 |  | 0.96(0.68,1.36) |  |  |  |
| Hepatobiliary system cancer | 4 | mix | Asia | All | fixed | 0.60 | 0.39 |  | 0.87(0.74,1.04) |  |  |  |
| Hepatobiliary system cancer | 10 | male | All | All | fixed | 34.70 | 0.13 |  | 0.93(0.80,1.09) |  |  |  |
| Hepatobiliary system cancer | 6 | mix | All | All | fixed | 0.00 | 0.94 |  | 0.81(0.60,1.10) |  |  |  |
| Hepatobiliary system cancer | 2 | mix | All | Chromate production workers | fixed | 0.00 | 0.32 |  | 1.05(0.63,1.75) |  |  |  |
| Hepatobiliary system cancer | 2 | mix | All | Cement production workers | fixed | 0.00 | 0.44 |  | 0.87(0.65,1.17) |  |  |  |
| Hepatobiliary system cancer | 2 | mix | All | Aircraft manufacturing workers | fixed | 0.00 | 0.66 |  | 0.91(0.63,1.32) |  |  |  |
| Hepatobiliary system cancer | 2 | mix | All | Chromium platers | fixed | 47.30 | 0.17 |  | 1.07(0.66,1.75) |  |  |  |
| Hepatobiliary system cancer | 2 | mix | All | Tanners | fixed | 0.00 | 0.88 |  | 0.68(0.28,1.67) |  |  |  |
| Hepatobiliary system cancer | 4 | mix | All | Welders | fixed | 0.00 | 0.84 |  | 0.82(0.66,1.01) |  |  |  |
| Hepatobiliary system cancer | 2 | mix | All | Painters | random | 50.10 | 0.16 |  | 2.23(1.14,4.37) |  |  |  |
| Pancreas cancer | 16 | mix | All | All | fixed | 0.00 | 0.91 | 0.19 | 0.94(0.81,1.08) |  |  |  |
| Pancreas cancer | 4 | mix | North America | All | fixed | 0.00 | 0.43 |  | 0.95(0.78,1.16) |  |  |  |
| Pancreas cancer | 11 | mix | Europe | All | fixed | 0.00 | 0.85 |  | 0.92(0.75,1.12) |  |  |  |
| Pancreas cancer | 7 | male | All | All | fixed | 0.00 | 0.64 |  | 0.95(0.74,1.21) |  |  |  |
| Pancreas cancer | 9 | mix | All | All | fixed | 0.00 | 0.85 |  | 0.93(0.79,1.10) |  |  |  |
| Pancreas cancer | 3 | mix | All | Chromate production workers | fixed | 0.00 | 0.53 |  | 0.75(0.49,1.15) |  |  |  |
| Pancreas cancer | 3 | mix | All | Cement production workers | fixed | 0.00 | 0.91 |  | 0.91(0.57,1.46) |  |  |  |
| Pancreas cancer | 2 | mix | All | Aircraft manufacturing workers | fixed | 0.00 | 0.85 |  | 1.05(0.82,1.34) |  |  |  |
| Pancreas cancer | 5 | mix | All | Tanners | fixed | 0.00 | 0.73 |  | 0.82(0.62,1.07) |  |  |  |
| Pancreas cancer | 2 | mix | All | Welders | fixed | 0.00 | 0.55 |  | 1.08(0.80,1.46) |  |  |  |
| Intestine cancer | 10 | male | All | All | fixed | 0.00 | 0.96 | 0.51 | 0.98(0.81,1.18) |  |  |  |
| Intestine cancer | 8 | male | Europe | All | fixed | 4.00 | 0.37 |  | 0.99(0.79,1.24) |  |  |  |
| Intestine cancer | 2 | male | All | Chromate production workers | fixed | 0.00 | 0.89 |  | 0.86(0.64,1.16) |  | |  |
| Intestine cancer | 9 | male | All | Aircraft manufacturing workers | fixed | 5.50 | 0.39 |  | 1.16(0.99,1.37) |  | |  |
| Intestine cancer | 4 | male | All | Tanners | fixed | 0.00 | 0.77 |  | 0.97(0.52,1.78) |  | |  |
| Intestine cancer | 4 | male | All | Welders | fixed | 0.00 | 0.45 |  | 0.92(0.73,1.17) |  |  |  |
| Colon cancer | 12 | mix | All | All | fixed | 0.00 | 0.69 | 0.28 | 1.06(0.93,1.21) |  |  |  |
| Colon cancer | 8 | mix | Europe | All | fixed | 0.00 | 0.53 |  | 0.99(0.81,1.22) |  |  |  |
| Colon cancer | 3 | mix | North America | All | fixed | 0.00 | 0.88 |  | 1.12(0.95,1.34) |  |  |  |
| Colon cancer | 8 | mix | All | All | fixed | 0.00 | 0.54 |  | 1.05(0.91,1.21) |  |  |  |
| Colon cancer | 4 | male | All | All | fixed | 0.00 | 0.54 |  | 1.11(0.79,1.58) |  |  |  |
| Colon cancer | 2 | mix | All | Cement production workers | fixed | 0.00 | 0.80 |  | 0.98(0.61,1.57) |  |  |  |
| Colon cancer | 2 | mix | All | Aircraft manufacturing workers | fixed | 0.00 | 0.62 |  | 1.12(0.94,1.35) |  |  |  |
| Colon cancer | 3 | mix | All | Tanners | fixed | 24.40 | 0.27 |  | 1.00(0.78,1.28) |  | |  |
| Colon cancer | 3 | mix | All | Welders | fixed | 16.20 | 0.30 |  | 1.16(0.77,1.75) |  |  |  |
| Rectum cancer | 23 | mix | All | All | fixed | 0.00 | 0.91 | 0.32 | 1.14(0.98,1.33) |  |  |  |
| Rectum cancer | 3 | mix | North America | All | fixed | 0.00 | 0.53 |  | 0.83(0.54,1.27) |  |  |  |
| Rectum cancer | 17 | mix | Europe | All | fixed | 0.00 | 0.92 |  | 1.20(1.01,1.42) |  |  |  |
| Rectum cancer | 3 | mix | Asia | All | fixed | 0.00 | 0.57 |  | 1.20(0.58,2.49) |  |  |  |
| Rectum cancer | 15 | male | All | All | fixed | 0.00 | 0.97 |  | 1.20(0.97,1.48) |  |  |  |
| Rectum cancer | 8 | mix | All | All | fixed | 6.40 | 0.38 |  | 1.08(0.86,1.35) |  |  |  |
| Rectum cancer | 2 | mix | All | Cement production workers | fixed | 0.00 | 0.80 |  | 0.98(0.61,1.57) |  |  |  |
| Rectum cancer | 2 | mix | All | Aircraft manufacturing workers | fixed | 0.00 | 0.62 |  | 1.12(0.94,1.35) |  |  |  |
| Rectum cancer | 3 | mix | All | Tanners | fixed | 24.40 | 0.27 |  | 1.00(0.78,1.28) |  | |  |
| Rectum cancer | 3 | mix | All | Welders | fixed | 16.20 | 0.30 |  | 1.16(0.77,1.75) |  |  |  |
| Urinary system cancer | 36 | mix | All | All | fixed | 35.00 | 0.02 | 0.12 | 1.20(1.07,1.35) |  |  |  |
| Urinary system cancer | 10 | mix | North America | All | fixed | 15.60 | 0.30 |  | 0.99(0.81,1.21) |  |  |  |
| Urinary system cancer | 25 | mix | Europe | All | fixed | 35.10 | 0.04 |  | 1.33(1.15,1.54) |  |  |  |
| Urinary system cancer | 19 | male | All | All | fixed | 28.40 | 0.12 |  | 1.39(1.18,1.65) |  |  |  |
| Urinary system cancer | 17 | mix | All | All | fixed | 29.60 | 0.12 |  | 1.04(0.89,1.23) |  |  |  |
| Urinary system cancer | 2 | mix | All | Chromate production workers | fixed | 0.00 | 0.72 |  | 0.73(0.43,1.25) |  |  |  |
| Urinary system cancer | 3 | mix | All | Cement production workers | fixed | 0.00 | 0.77 |  | 1.05(0.57,1.94) |  |  |  |
| Urinary system cancer | 4 | mix | All | Aircraft manufacturing workers | fixed | 0.00 | 0.54 |  | 0.97(0.74,1.27) |  |  |  |
| Urinary system cancer | 11 | mix | All | Tanners | fixed | 44.20 | 0.06 |  | 1.03(0.83,1.29) |  |  |  |
| Urinary system cancer | 10 | mix | All | Welders | fixed | 0.00 | 0.56 |  | 1.42(1.18,1.72) |  |  |  |
| Urinary system cancer | 2 | mix | All | Painters | fixed | 0.00 | 0.64 |  | 1.89(1.05,3.40) |  |  |  |
| Urinary system cancer | 3 | mix | All | Masons | fixed | 0.00 | 0.93 |  | 5.61(2.32,13.58) |  |  |  |
| Bladder cancer | 16 | mix | All | All | fixed | 35.90 | 0.08 | 0.27 | 1.24(1.05,1.47) |  |  |  |
| Bladder cancer | 13 | mix | Europe | All | fixed | 37.00 | 0.09 |  | 1.27(1.07,1.52) |  |  |  |
| Bladder cancer | 3 | mix | North America | All | fixed | 46.70 | 0.15 |  | 1.03(0.63,1.70) |  |  |  |
| Bladder cancer | 8 | mix | All | All | random | 56.80 | 0.02 |  | 1.13(0.90,1.44) |  |  |  |
| Bladder cancer | 8 | male | All | All | fixed | 0.00 | 0.54 |  | 1.36(1.08,1.72) |  |  |  |
| Bladder cancer | 6 | mix | All | Tanners | random | 52.10 | 0.06 |  | 1.01(0.78,1.32) |  |  |  |
| Bladder cancer | 7 | mix | All | Welders | fixed | 15.60 | 0.31 |  | 1.41(1.11,1.79) |  |  |  |
| Kidney cancer | 12 | mix | All | All | fixed | 6.10 | 0.39 | 0.50 | 1.15(0.91,1.45) |  |  |  |
| Kidney cancer | 7 | mix | Europe | All | fixed | 14.80 | 0.32 |  | 1.30(0.91,1.84) |  |  |  |
| Kidney cancer | 4 | mix | North America | All | fixed | 21.00 | 0.28 |  | 1.06(0.78,1.43) |  |  |  |
| Kidney cancer | 7 | mix | All | All | fixed | 0.00 | 0.68 |  | 0.94(0.71,1.25) |  |  |  |
| Kidney cancer | 5 | male | All | All | fixed | 0.00 | 0.70 |  | 1.68(1.14,2.47) |  |  |  |
| Kidney cancer | 2 | mix | All | Cement production workers | fixed | 0.00 | 0.74 |  | 1.06(0.44,2.55) |  |  |  |
| Kidney cancer | 2 | mix | All | Aircraft manufacturing workers | fixed | 0.00 | 0.47 |  | 0.92(0.61,1.39) |  |  |  |
| Kidney cancer | 5 | mix | All | Tanners | fixed | 45.90 | 0.12 |  | 1.08(0.72,1.62) |  |  |  |
| Kidney cancer | 2 | mix | All | Welders | fixed | 0.00 | 0.54 |  | 1.58(1.00,2.48) |  | |  |
| Lymphatic and Hematopoietic cancerCancer | 47 | mix | All | All | fixed | 10.20 | 0.28 | 0.53 | 1.03(0.93,1.13) |  |  |  |
| Lymphatic and Hematopoietic cancer | 14 | mix | North America | All | fixed | 24.90 | 0.19 |  | 0.97(0.85,1.10) |  |  |  |
| Lymphatic and Hematopoietic cancer | 30 | mix | Europe | All | fixed | 0.00 | 0.74 |  | 1.05(0.90,1.22) |  |  |  |
| Lymphatic and Hematopoietic cancer | 3 | mix | Asia | All | random | 61.10 | 0.08 |  | 1.57(1.04,2.37) |  |  |  |
| Lymphatic and Hematopoietic cancer | 24 | male | All | All | fixed | 0.00 | 0.51 |  | 0.99(0.85,1.16) |  |  |  |
| Lymphatic and Hematopoietic cancer | 23 | mix | All | All | fixed | 23.30 | 0.15 |  | 1.05(0.93,1.19) |  |  |  |
| Lymphatic and Hematopoietic cancer | 7 | mix | All | Chromate production workers | fixed | 0.40 | 0.42 |  | 0.93(0.73,1.19) |  |  |  |
| Lymphatic and Hematopoietic cancer | 2 | mix | All | Cement production workers | random | 58.40 | 0.12 |  | 1.25(0.80,1.96) |  |  |  |
| Lymphatic and Hematopoietic cancer | 10 | mix | All | Aircraft manufacturing workers | fixed | 0.00 | 0.67 |  | 1.10(0.94,1.29) |  |  |  |
| Lymphatic and Hematopoietic cancer | 10 | mix | All | Tanners | fixed | 40.70 | 0.09 |  | 0.93(0.75,1.15) |  |  |  |
| Lymphatic and Hematopoietic cancer | 14 | mix | All | Welders | fixed | 0.00 | 0.70 |  | 0.97(0.78,1.20) |  |  |  |
| Lymphatic and Hematopoietic cancer | 2 | mix | All | Painters | fixed | 0.00 | 0.78 |  | 0.54(0.19,1.51) |  |  |  |
| Leukemia | 16 | mix | All | All | fixed | 1.10 | 0.44 |  | 1.00(0.86,1.16) |  | |  |
| Leukemia | 6 | mix | North America | All | fixed | 0.00 | 0.90 |  | 0.94(0.77,1.15) |  |  |  |
| Leukemia | 9 | mix | Europe | All | fixed | 27.10 | 0.20 |  | 0.99(0.76,1.29) |  |  |  |
| Leukemia | 7 | male | All | All | fixed | 0.00 | 0.45 |  | 1.06(0.82,1.37) |  |  |  |
| Leukemia | 9 | mix | All | All | fixed | 11.80 | 0.34 |  | 0.96(0.80,1.16) |  |  |  |
| Leukemia | 4 | mix | All | Aircraft manufacturing workers | fixed | 0.00 | 0.98 |  | 0.96(0.76,1.21) |  |  |  |
| Leukemia | 5 | mix | All | Tanners | fixed | 45.80 | 0.12 |  | 1.06(0.78,1.44) |  |  |  |
| Leukemia | 5 | mix | All | Welders | fixed | 30.70 | 0.22 |  | 1.01(0.75,1.38) |  |  |  |
| Lymphoma | 19 | mix | All | All | fixed | 15.20 | 0.27 |  | 1.15(0.96,1.39) |  |  |  |
| Non-Hodgkin’s lymphoma | 7 | mix | All | All | fixed | 18.40 | 0.29 |  | 1.07(0.84,1.37) |  |  |  |
| Hodgkin's lymphoma | 6 | mix | All | All | fixed | 0.00 | 0.43 |  | 1.40(0.87,2.25) |  |  |  |
| Multiple myeloma | 7 | mix | All | All | fixed | 0.00 | 0.64 |  | 1.10(0.80,1.50) |  | |  |
| Genitourinary cancerm cancer | 27 | mix | All | All | fixed | 5.10 | 0.39 | 0.71 | 1.04(0.93,1.17) |  |  |  |
| Genitourinary cancer | 11 | mix | North America | All | fixed | 19.20 | 0.26 |  | 1.08(0.94,1.24) |  |  |  |
| Genitourinary cancer | 16 | mix | Europe | All | fixed | 0.00 | 0.51 |  | 0.96(0.79,1.18) |  |  |  |
| Genitourinary cancer | 17 | male | All | All | fixed | 26.00 | 0.16 |  | 1.03(0.91,1.16) |  |  |  |
| Genitourinary cancer | 7 | female | All | All | fixed | 0.00 | 0.78 |  | 1.15(0.82,1.62) |  |  |  |
| Genitourinary cancer | 3 | mix | All | All | fixed | 5.80 | 0.35 |  | 1.00(0.53,1.89) |  | |  |
| Genitourinary cancer | 4 | mix | All | Chromate production workers | fixed | 4.00 | 0.37 |  | 0.86(0.64,1.16) |  |  |  |
| Genitourinary cancer | 9 | mix | All | Aircraft manufacturing workers | fixed | 5.50 | 0.39 |  | 1.16(0.99,1.37) |  |  |  |
| Genitourinary cancer | 4 | mix | All | Tanners | fixed | 0.00 | 0.77 |  | 0.97(0.52,1.78) |  |  |  |
| Genitourinary cancer | 6 | mix | All | Welders | fixed | 0.00 | 0.45 |  | 0.92(0.73,1.17) |  |  |  |
| Genitourinary cancer | 2 | mix | All | Painters | random | 77.70 | 0.03 |  | 1.31(0.63,2.69) |  |  |  |
| Breast cancer | 4 | female | All | All | fixed | 0.00 | 0.48 |  | 1.12(0.76,1.65) |  |  |  |
| Prostate cancer | 14 | male | All | All | fixed | 0.00 | 0.75 |  | 0.99(0.87,1.12) |  |  |  |
| Prostate cancer | 3 | male | North America | All | fixed | 28.00 | 0.25 |  | 1.03(0.88,1.20) |  |  |  |
| Prostate cancer | 11 | male | Europe | All | fixed | 0.00 | 0.84 |  | 0.91(0.73,1.14) |  |  |  |
| Prostate cancer | 3 | male | All | Chromate production workers | fixed | 0.00 | 0.48 |  | 0.89(0.66,1.19) |  |  |  |
| Prostate cancer | 5 | male | All | Welders | fixed | 0.00 | 0.43 |  | 0.89(0.69,1.15) |  |  |  |
| Prostate cancer | 2 | male | All | Tanners | fixed | 0.00 | 0.53 |  | 0.87(0.31,2.46) |  |  |  |
| Testis cancer | 4 | male | All | All | fixed | 16.90 | 0.31 |  | 2.55(1.38,4.71) |  |  |  |
| Bone cancer | 5 | mix | All | All | fixed | 0.00 | 0.42 |  | 2.06(1.12,3.81) |  |  |  |
| Thyroid cancer | 3 | mix | All | All | fixed | 31.20 | 0.23 |  | 2.41(1.19,4.87) |  |  |  |
| Buccal cavity and pharynx cancercer | 17 | mix | All | All | fixed | 3.10 | 0.42 |  | 0.91(0.75,1.10) |  |  |  |
| Buccal cavity and pharynx cancercer | 12 | male | All | All | fixed | 0.00 | 0.64 |  | 1.07(0.84,1.38) |  |  |  |
| Buccal cavity and pharynx cancercer | 5 | mix | All | All | fixed | 0.00 | 0.47 |  | 0.72(0.54,0.97) |  |  |  |
| Buccal cavity and pharynx cancercer | 5 | mix | North America | All | random | 52.00 | 0.08 |  | 0.97(0.69,1.36) |  |  |  |
| Buccal cavity and pharynx cancercer | 12 | mix | Europe | All | fixed | 0.00 | 0.72 |  | 0.88(0.70,1.11) |  |  |  |
| Buccal cavity and pharynx cancercer | 4 | mix | All | Chromate production workers | fixed | 0.00 | 0.73 |  | 1.37(0.87,2.15) |  |  |  |
| Buccal cavity and pharynx cancercer | 2 | mix | All | Aircraft manufacturing workers | fixed | 47.00 | 0.17 |  | 0.62(0.37,1.02) |  |  |  |
| Buccal cavity and pharynx cancercer | 8 | mix | All | Welders | fixed | 0.00 | 0.97 |  | 0.83(0.63,1.09) |  |  |  |
| Brain and central nervous system cancer | 9 | mix | All | All | random | 62.40 | 0.01 |  | 1.22(0.67,2.23) |  |  |  |
| Brain and central nervous system cancer | 5 | male | All | All | random | 78.90 | 0.00 |  | 1.68(0.49,5.76) |  |  |  |
| Brain and central nervous system cancer | 4 | mix | All | All | fixed | 0.00 | 0.54 |  | 0.91(0.61,1.36) |  |  |  |
| Brain and central nervous system cancer | 2 | mix | Asia | All | fixed | 73.60 | 0.05 |  | 3.89(1.57,9.67) |  |  |  |
| Brain and central nervous system cancer | 2 | mix | North America | All | fixed | 0.00 | 0.37 |  | 0.82(0.53,1.27) |  |  |  |
| Brain and central nervous system cancer | 5 | mix | Europe | All | fixed | 37.30 | 0.17 |  | 0.78(0.49,1.25) |  |  |  |
| Brain cancer | 6 | mix | All | All | random | 74.50 | 0.00 |  | 1.67(0.62,4.46) |  |  |  |
| Skin cancer | 6 | mix | All | All | fixed | 8.00 | 0.37 |  | 0.99(0.66,1.48) |  |  |  |
| Melanoma | 3 | mix | All | All | fixed | 0.00 | 0.42 |  | 0.90(0.52,1.54) |  |  |  |
| Connective and other soft tissue cancere cancer | 3 | mix | All | All | fixed | 0.00 | 0.53 |  | 1.22(0.62,2.41) |  |  |  |
| Other cancers | 23 | mix | All | All | random | 71.50 | 0.00 |  | 1.22(0.98,1.51) |  |  |  |
| **Abbreviations:** |  |  |  |  |  |  |  |  |  |  |  |  |
|  |  |  |  |  |  |  |  |  |  |  |  |  |

I^2^: the percentage of total variation across studies due to heterogeneity rather than chance; SMR: standardized mortality ratio; CI: confidence interval
