# Supplementary material for: The Effect of Hexavalent Chromium on the Incidence and Mortality of Human Cancers: A Meta-Analysis Based on Published Epidemiological Cohort Studies
Source: Front Oncol. 2019 Feb 4;9:24. doi: 10.3389/fonc.2019.00024 (PMC6369173; doi:10.3389/fonc.2019.00024)
Supplement: Supplementary file 2 [file Table_2.DOCX]

**The effect of hexavalent chromium on the incidence and mortality of human cancers: A meta-analysis based on published epidemiological cohort studies**

**Authors:** Yujiao Deng1,2,†, Meng Wang1,2,†, Tian Tian1,2,†, Shuai Lin1, Peng Xu1, Linghui Zhou1, Cong Dai1, Qian Hao1, Ying Wu1, Zhen Zhai1, Yue Zhu1, Guihua Zhuang3, and Zhijun Dai1,2*

***Correspondence to:** Zhijun Dai, (E-Mail: dzj0911@126.com), or Guihua Zhuang, (E-Mail: [zhuanggh@xjtu.edu.cn](mailto:zhuanggh@xjtu.edu.cn)).

**Supplementary table 2. The results of all subgroup analysis for standardized incidence ratio (SIR)**

| **Cancer Type** | **No of studystudy** | | **Sex** | | **District** | | **Profession** | | **Model Type** | | **I^2^ (%)** | **P value** | | **Egger test (P value) value)** | | | **SIR(CIs)** | | | |  |
| --- | --- | --- | --- | --- | --- | --- | --- | --- | --- | --- | --- | --- | --- | --- | --- | --- | --- | --- | --- | --- | --- |
| All types of cancer | 17 | | mix | | All | | All | | random | | 39.40 | 0.05 | | 0.09 | | | 1.06(1.04,1.09) | | | |  |
| All types of cancer | 2 | | mix | | North America | | All | | random | | 81.00 | 0.02 | |  | | | 1.09(0.94,1.26) | | | |  |
| All types of cancer | 14 | | mix | | Europe | | All | | fixed | | 36.90 | 0.08 | |  | | | 1.06(1.04,1.09) | | | |  |
| All types of cancer | 6 | | mix | | All | | All | | fixed | | 48.80 | 0.08 | |  | | | 1.06(1.00,1.12) | | | |  |
| All types of cancer | 10 | | male | | All | | All | | fixed | | 44.30 | 0.06 | |  | | | 1.05(0.99,1.10) | | | |  |
| All types of cancer | 9 | | mix | | All | | Welders | | fixed | | 40.60 | 0.09 | |  | | | 1.03(0.99,1.08) | | | |  |
| All types of cancer | 6 | | mix | | All | | Cement Industry Workers | | fixed | | 28.50 | 0.24 | |  | | | 1.07(1.04,1.10) | | | |  |
| Respiratory system cancer | 30 | | mix | | All | | All | | fixed | | 5.80 | 0.38 | | 0.98 | | | 1.27(1.19,1.36) | | | |  |
| Respiratory system cancer | 8 | | mix | | All | | All | | fixed | | 15.80 | 0.31 | |  | | | 1.17(0.99,1.38) | | | |  |
| Respiratory system cancer | 21 | | male | | All | | All | | fixed | | 4.80 | 0.40 | |  | | | 1.29(1.20,1.38) | | | |  |
| Respiratory system cancer | 26 | | mix | | Europe | | All | | fixed | | 10.50 | 0.31 | |  | | | 1.27(1.19,1.36) | | | |  |
| Respiratory system cancer | 3 | | mix | | North America | | All | | fixed | | 0.00 | 0.68 | |  | | | 1.45(1.08,1.94) | | | |  |
| Respiratory system cancer | 17 | | mix | | All | | Welders | | fixed | | 30.70 | 0.11 | |  | | | 1.29(1.15,1.45) | | | |  |
| Respiratory system cancer | 11 | | mix | | All | | Cement Industry Workers | | fixed | | 0.00 | 0.71 | |  | | | 1.26(1.17,1.37) | | | |  |
| Respiratory system cancer | 2 | | mix | | All | | Tanners | | fixed | | 0.00 | 0.49 | |  | | | 1.27(0.89,1.82) | | | |  |
| Nasal cancer | 3 | | mix | | All | | All | | fixed | | 0.00 | 0.99 | |  | | | 2.14(0.79,5.80) | | | |  |
| Lung cancer | 14 | | mix | | All | | All | | fixed | | 35.20 | 0.09 | | 0.84 | | | 1.28(1.20,1.37) | | | |  |
| Lung cancer | 9 | | male | | All | | All | | fixed | | 28.60 | 0.19 | |  | | | 1.31(1.21,1.41) | | | |  |
| Lung cancer | 4 | | mix | | All | | All | | random | | 55.60 | 0.08 | |  | | | 1.15(0.97,1.36) | | | |  |
| Lung cancer | 11 | | mix | | Europe | | All | | fixed | | 45.20 | 0.05 | |  | | | 1.28(1.19,1.37) | | | |  |
| Lung cancer | 2 | | mix | | North America | | All | | fixed | | 0.00 | 0.83 | |  | | | 1.52(1.11,2.07) | | | |  |
| Lung cancer | 6 | | mix | | All | | Cement Industry Workers | | fixed | | 0.00 | 0.53 | |  | | | 1.27(1.17,1.38) | | | |  |
| Lung cancer | 7 | | mix | | All | | welders | | random | | 62.00 | 0.02 | |  | | | 1.31(1.16,1.47) | | | |  |
| Larynx cancer | 10 | | mix | | All | | All | | fixed | | 0.00 | 0.99 | | 0.86 | | | 1.14(0.89,1.45) | | | |  |
| Larynx cancer | 9 | | male | | All | | All | | fixed | | 0.00 | 0.97 | |  | | | 1.13(0.88,1.46) | | | |  |
| Larynx cancer | 8 | | mix | | Europe | | All | | fixed | | 0.00 | 0.96 | |  | | | 1.16(0.89,1.50) | | | |  |
| Larynx cancer | 5 | | mix | | All | | Cement Industry Workers | | fixed | | 0.00 | 0.94 | |  | | | 1.13(0.85,1.48) | | | |  |
| Larynx cancer | 5 | | mix | | All | | Welders | | fixed | | 0.00 | 0.94 | |  | | | 1.18(0.68,2.04) | | | |  |
| Pleural mesothelioma | 3 | | mix | | All | | All | | fixed | | 0.00 | 0.76 | |  | | | 1.73(1.08,2.77) | | | |  |
| Thyroid cancer | 4 | | mix | | All | | All | | fixed | | 0.00 | 0.41 | |  | | | 0.81(0.54,1.21) | | | |  |
| Bone cancer | 2 | | mix | | All | | All | | fixed | | 0.00 | 0.80 | |  | | | 1.64(0.86,3.16) | | | |  |
| Buccal cavity and pharynx cancer | 16 | | mix | | All | | All | | fixed | | 4.30 | 0.40 | | 0.13 | | | 1.30(1.11,1.54) | | | |  |
| Buccal cavity and pharynx cancer | 15 | | male | | All | | All | | fixed | | 9.80 | 0.34 | |  | | | 1.32(1.11,1.56) | | | |  |
| Buccal cavity and pharynx cancer | 11 | | mix | | Europe | | All | | fixed | | 35.40 | 0.12 | |  | | | 1.29(1.09,1.54) | | | |  |
| Buccal cavity and pharynx cancer | 4 | | mix | | Asia | | All | | fixed | | 0.00 | 0.99 | |  | | | 1.44(0.75,2.77) | | | |  |
| Buccal cavity and pharynx cancer | 4 | | mix | | All | | Welders | | fixed | | 0.00 | 0.88 | |  | | | 1.43(1.01,2.02) | | | |  |
| Buccal cavity and pharynx cancer | 12 | | mix | | All | | Cement Industry Workers | | fixed | | 24.80 | 0.20 | |  | | | 1.27(1.05,1.53) | | | |  |
| Urinary cancer | 23 | | mix | | All | | All | | fixed | | 0.00 | 0.60 | | 0.47 | | | 1.03(0.95,1.13) | | | |  |
| Urinary cancer | 5 | | mix | | All | | All | | fixed | | 0.00 | 0.41 | |  | | | 0.87(0.65,1.16) | | | |  |
| Urinary cancer | 16 | | male | | All | | All | | fixed | | 0.00 | 0.54 | |  | | | 1.05(0.96,1.15) | | | |  |
| Urinary cancer | 2 | | female | | All | | All | | fixed | | 0.00 | 0.70 | |  | | | 1.41(0.46,4.31) | | | |  |
| Urinary cancer | 16 | | mix | | Europe | | All | | fixed | | 0.00 | 0.50 | |  | | | 1.03(0.94,1.12) | | | |  |
| Urinary cancer | 3 | | mix | | Asia | | All | | fixed | | 0.00 | 0.38 | |  | | | 0.85(0.48,1.52) | | | |  |
| Urinary cancer | 4 | | mix | | North America | | All | | fixed | | 0.00 | 0.75 | |  | | | 1.44(0.89,2.35) | | | |  |
| Urinary cancer | 10 | | mix | | All | | Welders | | fixed | | 24.90 | 0.21 | |  | | | 1.04(0.82,1.31) | | | |  |
| Urinary cancer | 11 | | mix | | All | | Cement Industry Workers | | fixed | | 0.00 | 0.69 | |  | | | 1.04(0.95,1.14) | | | |  |
| Urinary cancer | 2 | | mix | | All | | Tanners | | fixed | | 0.00 | 0.78 | |  | | | 0.93(0.64,1.36) | | | |  |
| Bladder cancer | 9 | | mix | | All | | All | | fixed | | 0.00 | 0.66 | | 0.44 | | | 1.09(0.98,1.22) | | | |  |
| Bladder cancer | 2 | | mix | | All | | All | | fixed | | 0.00 | 0.91 | |  | | | 0.97(0.70,1.36) | | | |  |
| Bladder cancer | 6 | | male | | All | | All | | fixed | | 1.60 | 0.41 | |  | | | 1.11(0.99,1.24) | | | |  |
| Bladder cancer | 6 | | mix | | Europe | | All | | fixed | | 0.00 | 0.64 | |  | | | 1.08(0.97,1.20) | | | |  |
| Bladder cancer | 2 | | mix | | North America | | All | | fixed | | 0.00 | 0.96 | |  | | | 1.81(0.96,3.43) | | | |  |
| Bladder cancer | 4 | | mix | | All | | Welders | | fixed | | 0.00 | 0.53 | |  | | | 1.22(0.93,1.60) | | | |  |
| Bladder cancer | 4 | | mix | | All | | Cement Industry Workers | | fixed | | 0.00 | 0.45 | |  | | | 1.08(0.96,1.22) | | | |  |
| Kidney cancer | 10 | | mix | | All | | All | | fixed | | 0.00 | 0.49 | | 0.40 | | | 0.93(0.79,1.08) | | | |  |
| Kidney cancer | 3 | | mix | | All | | All | | fixed | | 9.60 | 0.33 | |  | | | 0.63(0.36,1.11) | | | |  |
| Kidney cancer | 6 | | male | | All | | All | | fixed | | 0.00 | 0.52 | |  | | | 0.95(0.81,1.12) | | | |  |
| Kidney cancer | 7 | | mix | | Europe | | All | | fixed | | 13.60 | 0.33 | |  | | | 0.93(0.79,1.09) | | | |  |
| Kidney cancer | 2 | | mix | | North America | | All | | fixed | | 0.00 | 0.83 | |  | | | 1.06(0.50,2.44) | | | |  |
| Kidney cancer | 4 | | mix | | All | | Welders | | fixed | | 0.00 | 0.56 | |  | | | 0.54(0.30,1.00) | | | |  |
| Kidney cancer | 5 | | mix | | All | | Cement Industry Workers | | fixed | | 0.00 | 0.60 | |  | | | 0.97(0.82,1.44) | | | |  |
| Brain cancer | 3 | | mix | | All | | All | | fixed | | 0.00 | 0.68 | |  | | | 1.04(0.87,1.24) | | | |  |
| Skin cancer | 12 | | mix | | All | | All | | fixed | | 25.50 | 0.19 | | 0.64 | | | 1.02(0.95,1.10) | | | |  |
| Skin cancer | 4 | | mix | | All | | All | | fixed | | 47.60 | 0.13 | |  | | | 1.07(0.98,1.16) | | | |  |
| Skin cancer | 7 | | male | | All | | All | | fixed | | 3.60 | 0.40 | |  | | | 0.93(0.82,1.06) | | | |  |
| Skin cancer | 8 | | mix | | Europe | | All | | fixed | | 44.50 | 0.08 | |  | | | 1.02(0.95,1.09) | | | |  |
| Skin cancer | 3 | | mix | | North America | | All | | fixed | | 0.00 | 0.67 | |  | | | 1.29(0.84,2.00) | | | |  |
| Skin cancer | 5 | | mix | | All | | Welders | | fixed | | 34.60 | 0.19 | |  | | | 1.07(0.98,1.16) | | | |  |
| Skin cancer | 7 | | mix | | All | | Cement Industry Workers | | fixed | | 0.00 | 0.47 | |  | | | 0.93(0.82,1.06) | | | |  |
| Melanoma | 4 | | mix | | All | | All | | random | | 61.30 | 0.05 | |  | | | 0.92(0.77,1.11) | | | |  |
| Soft tissue cancer | 3 | | mix | | All | | All | | fixed | | 44.90 | 0.16 | |  | | | 1.20(0.84,1.71) | | | |  |
| Female genital system cancer | 8 | | female | | All | | All | | fixed | | 20.20 | 0.27 | | 0.25 | | | 1.02(0.85,1.23) | | | |  |
| Female genital system cancer | 4 | | female | | All | | Welders | | fixed | | 0.00 | 0.61 | |  | | | 1.10(0.84,1.44) | | | |  |
| Female genital system cancer | 2 | | female | | All | | Cement Industry Workers | | fixed | | 0.00 | 0.54 | |  | | | 0.72(0.46,1.12) | | | |  |
| Female genital system cancer | 6 | | female | | Europe | | All | | fixed | | 9.20 | 0.36 | |  | | | 1.10(0.90,1.34) | | | |  |
| Female genital system cancer | 2 | | female | | North America | | All | | fixed | | 0.00 | 0.54 | |  | | | 0.72(0.46,1.12) | | | |  |
| Breast cancer | 3 | | female | | All | | All | | fixed | | 48.70 | 0.14 | |  | | | 1.08(0.84,1.38) | | | |  |
| Male genital system cancer | 13 | | male | | All | | All | | fixed | | 51.10 | 0.02 | | 0.29 | | | 1.14(1.07,1.21) | | | |  |
| Male genital system cancer | 10 | | male | | Europe | | All | | random | | 62.30 | 0.01 | |  | | | 1.14(1.07,1.21) | | | |  |
| Male genital system cancer | 2 | | male | | Asia | | All | | fixed | | 0.00 | 0.86 | |  | | | 1.52(0.81,2.86) | | | |  |
| Male genital system cancer | 5 | | male | | All | | Welders | | fixed | | 0.00 | 0.52 | |  | | | 1.42(1.22,1.65) | | | |  |
| Male genital system cancer | 7 | | male | | All | | Cement Industry Workers | | fixed | | 20.60 | 0.27 | |  | | | 1.07(1.01,1.15) | | | |  |
| Prostate cancer | 9 | | male | | All | | All | | random | | 52.10 | 0.03 | | 0.02 | | | 1.15(1.08,1.22) | | | |  |
| Prostate cancer | 4 | | male | | All | | Welders | | fixed | | 0.00 | 0.61 | |  | | | 1.44(1.23,1.67) | | | |  |
| Prostate cancer | 4 | | male | | All | | Cement Industry Workers | | fixed | | 0.00 | 0.83 | |  | | | 1.08(1.01,1.16) | | | |  |
| Prostate cancer | 8 | | male | | Europe | | All | | random | | 62.80 | 0.01 | |  | | | 1.15(1.08,1.22) | | | |  |
| Digestive cancer | 51 | | mix | | All | | All | | fixed | | 19.30 | 0.12 | | 0.63 | | | 1.05(1.00,1.11) | | | |  |
| Digestive cancer | 14 | | mix | | All | | All | | fixed | | 0.00 | 0.98 | |  | | | 1.08(0.91,1.28) | | | |  |
| Digestive cancer | 34 | | male | | All | | All | | fixed | | 42.00 | 0.01 | |  | | | 1.05(0.99,1.11) | | | |  |
| Digestive cancer | 3 | | female | | All | | All | | fixed | | 0.00 | 0.99 | |  | | | 0.85(0.41,1.77) | | | |  |
| Digestive cancer | 43 | | mix | | Europe | | All | | fixed | | 28.20 | 0.045 | |  | | | 1.06(1.00,1.12) | | | |  |
| Digestive cancer | 7 | | mix | | North America | | All | | fixed | | 0.00 | 0.91 | |  | | | 0.87(0.61,1.24) | | | |  |
| Digestive cancer | 25 | | mix | | All | | Welders | | fixed | | 0.00 | 0.77 | |  | | | 1.07(0.94,1.23) | | | |  |
| Digestive cancer | 22 | | mix | | All | | Cement Industry Workers | | random | | 50.50 | 0.01 | |  | | | 1.05(0.99,1.12) | | | |  |
| Digestive cancer | 4 | | mix | | All | | Tanners | | fixed | | 0.00 | 0.88 | |  | | | 1.02(0.78,1.32) | | | |  |
| Esophagus cancer | 5 | | mix | | All | | All | | fixed | | 0.00 | 0.94 | | 0.18 | | | 0.99(0.76,1.30) | | | |  |
| Esophagus cancer | 4 | | male | | All | | All | | fixed | | 0.00 | 0.87 | |  | | | 1.00(0.76,1.33) | | | |  |
| Esophagus cancer | 4 | | mix | | Europe | | All | | fixed | | 0.00 | 0.90 | |  | | | 1.01(0.76,1.33) | | | |  |
| Esophagus cancer | 2 | | mix | | All | | Welders | | fixed | | 0.00 | 0.68 | |  | | | 0.76(0.30,1.90) | | | |  |
| Esophagus cancer | 3 | | mix | | All | | Cement Industry Workers | | fixed | | 0.00 | 0.89 | |  | | | 1.02(0.77,1.36) | | | |  |
| Bowel cancer | 30 | | mix | | All | | All | | fixed | | 35.00 | 0.03 | | 0.03 | | | 1.03(0.96,1.12) | | | |  |
| Bowel cancer | 6 | | mix | | All | | All | | fixed | | 0.00 | 0.88 | |  | | | 1.12(0.89,1.41) | | | |  |
| Bowel cancer | 22 | | male | | All | | All | | fixed | | 50.40 | 0.04 | |  | | | 1.02(0.94,1.11) | | | |  |
| Bowel cancer | 2 | | female | | All | | All | | fixed | | 0.00 | 1.00 | |  | | | 0.90(0.32,2.50) | | | |  |
| Bowel cancer | 24 | | mix | | Europe | | All | | fixed | | 46.00 | 0.01 | |  | | | 1.03(0.95,1.12） | | | |  |
| Bowel cancer | 2 | | mix | | Asia | | All | | fixed | | 0.00 | 0.35 | |  | | | 1.10(0.72,1.70） | | | |  |
| Bowel cancer | 4 | | mix | | North America | | All | | fixed | | 0.00 | 0.78 | |  | | | 1.06(0.62,1.83） | | | |  |
| Bowel cancer | 15 | | mix | | All | | Welders | | fixed | | 0.00 | 0.48 | |  | | | 1.32(1.12,1.55) | | | |  |
| Bowel cancer | 13 | | mix | | All | | Cement Industry Workers | | fixed | | 38.70 | 0.08 | |  | | | 0.95(0.87,1.05) | | | |  |
| Colon cancer | 11 | | mix | | All | | All | | fixed | | 29.80 | 0.16 | | 0.30 | | | 1.17(0.96,1.42) | | | |  |
| Colon cancer | 3 | | mix | | All | | All | | fixed | | 0.00 | 0.70 | |  | | | 1.14(0.85,1.54) | | | |  |
| Colon cancer | 7 | | male | | All | | All | | random | | 55.10 | 0.04 | |  | | | 1.20(0.91,1.56) | | | |  |
| Colon cancer | 9 | | mix | | Europe | | All | | fixed | | 40.10 | 0.10 | |  | | | 1.19(0.97,1.46) | | | |  |
| Colon cancer | 2 | | mix | | North America | | All | | fixed | | 0.00 | 0.79 | |  | | | 0.77(0.31,1.92) | | | |  |
| Colon cancer | 6 | | mix | | All | | Welders | | fixed | | 29.80 | 0.16 | |  | | | 1.24(0.94,1.62) | | | |  |
| Colon cancer | 4 | | mix | | All | | Cement Industry Workers | | fixed | | 46.10 | 0.10 | |  | | | 1.17(0.79,1.72 | | | |  |
| Rectum cancer | 14 | | mix | | All | | All | | fixed | | 0.00 | 0.97 | | 0.03 | | | 1.09(0.96,1.22) | | | |  |
| Rectum cancer | 3 | | mix | | All | | All | | fixed | | 0.00 | 0.61 | |  | | | 1.08(0.75,1.55) | | | |  |
| Rectum cancer | 10 | | male | | All | | All | | fixed | | 0.00 | 0.89 | |  | | | 1.09(0.96,1.24) | | | |  |
| Rectum cancer | 11 | | mix | | Europe | | All | | fixed | | 0.00 | 0.93 | |  | | | 1.07(0.95,1.21) | | | |  |
| Rectum cancer | 2 | | mix | | North America | | All | | fixed | | 0.00 | 0.60 | |  | | | 1.27(0.64,2.51) | | | |  |
| Rectum cancer | 7 | | mix | | All | | Welders | | fixed | | 0.00 | 0.91 | |  | | | 1.25(0.97,1.62) | | | |  |
| Rectum cancer | 6 | | mix | | All | | Cement Industry Workers | | fixed | | 0.00 | 0.88 | |  | | | 1.05(0.91,1.21) | | | |  |
| Pancreas cancer | 8 | | mix | | All | | All | | fixed | | 0.00 | 0.95 | | 0.20 | | | 1.04(0.89,1.23) | | | |  |
| Pancreas cancer | 2 | | mix | | All | | All | | fixed | | 0.00 | 0.91 | |  | | | 1.24(0.83,1.86) | | | |  |
| Pancreas cancer | 6 | | male | | All | | All | | fixed | | 0.00 | 0.94 | |  | | | 1.01(0.84,1.20) | | | |  |
| Pancreas cancer | 6 | | mix | | Europe | | All | | fixed | | 0.00 | 0.85 | |  | | | 1.04(0.88,1.23) | | | |  |
| Pancreas cancer | 2 | | mix | | All | | Welders | | fixed | | 0.00 | 0.62 | |  | | | 1.17(0.71,1.92) | | | |  |
| Pancreas cancer | 5 | | mix | | All | | Cement Industry Workers | | fixed | | 0.00 | 0.88 | |  | | | 1.01(0.84,1.21) | | | |  |
| Stomach cancer | 14 | | mix | | All | | All | | fixed | | 43.30 | 0.04 | | 0.01 | | | 1.20(1.08,1.32) | | | |  |
| Stomach cancer | 3 | | mix | | All | | All | | fixed | | 0.00 | 0.92 | |  | | | 0.87(0.59,1.29) | | | |  |
| Stomach cancer | 10 | | male | | All | | All | | random | | 53.70 | 0.02 | |  | | | 1.23(1.11,1.36) | | | |  |
| Stomach cancer | 11 | | mix | | Europe | | All | | fixed | | 47.00 | 0.04 | |  | | | 1.21(1.09,1.35) | | | |  |
| Stomach cancer | 7 | | mix | | All | | Welders | | fixed | | 0.00 | 0.96 | |  | | | 0.75(0.57,1.00) | | | |  |
| Stomach cancer | 6 | | mix | | All | | Cement Industry Workers | | fixed | | 33.40 | 0.18 | |  | | | 1.30(1.17,1.45) | | | |  |
| Hepatobiliary system cancer | 4 | | mix | | All | | All | | fixed | | 1.70 | 0.38 | |  | | | 0.92(0.76,1.11） | | | |  |
| Hepatobiliary system cancer | 3 | | mix | | Europe | | All | | fixed | | 0.00 | 0.65 | |  | | | 1.11(0.50,2.45) | | | |  |
| Hepatobiliary system cancer | 2 | | mix | | All | | All | | fixed | | 0.00 | 0.38 | |  | | | 0.99(0.80,1.23） | | | |  |
| Hepatobiliary system cancer | 2 | | male | | All | | All | | random | | 51.10 | 0.15 | |  | | | 0.70(0.47,1.05） | | | |  |
| Hepatobiliary system cancer | 2 | | mix | | All | | Cement Industry Workers | | random | | 51.10 | 0.15 | |  | | | 0.91(0.74,1.10) | | | |  |
| Lymphatic and hematopoietic cancer | 24 | | mix | | All | | All | | fixed | | 0.00 | 0.99 | | 0.88 | | | 1.10(1.00,1.22) | | | |  |
| Lymphatic and hematopoietic cancer | 7 | | mix | | All | | All | | fixed | | 0.00 | 0.87 | |  | | | 1.29(1.00,1.67) | | | |  |
| Lymphatic and hematopoietic cancer | 16 | | male | | All | | All | | fixed | | 0.00 | 0.99 | |  | | | 1.08(0.97,1.20) | | | |  |
| Lymphatic and hematopoietic cancer | 19 | | mix | | Europe | | All | | fixed | | 0.00 | 0.99 | |  | | | 1.10(1.00,1.22) | | | |  |
| Lymphatic and hematopoietic cancer | 3 | | mix | | Asia | | All | | fixed | | 0.00 | 0.57 | |  | | | 1.03(0.54,1.96) | | | |  |
| Lymphatic and hematopoietic cancer | 2 | | mix | | North America | | All | | fixed | | 0.00 | 0.48 | |  | | | 1.07(0.58,1.98) | | | |  |
| Lymphatic and hematopoietic cancer | 10 | | mix | | All | | Welders | | fixed | | 0.00 | 0.87 | |  | | | 1.16(0.92,1.46) | | | |  |
| Lymphatic and hematopoietic cancer | 12 | | mix | | All | | Cement Industry Workers | | fixed | | 0.00 | 0.99 | |  | | | 1.07(0.96,1.20) | | | |  |
| Lymphatic and hematopoietic cancer | 2 | | mix | | All | | Tanners | | fixed | | 0.00 | 0.69 | |  | | | 1.45(0.86,2.43) | | | |  |
| Lymphoma | 5 | | mix | | All | | All | | fixed | | 0.00 | 0.79 | |  | | | 1.12(0.96,1.30) | | | |  |
| Hodgkin lymphoma | 2 | | mix | | All | | All | | fixed | | 4.50 | 0.31 | |  | | | 0.99(0.65,1.52) | | | |  |
| Non-Hodgkin lymphoma | 3 | | mix | | All | | All | | fixed | | 0.00 | 0.87 | |  | | | 1.14(0.97,1.34) | | | |  |
| Multiple myeloma | 5 | | mix | | All | | All | | fixed | | 0.00 | 0.75 | | 0.10 | | | 1.13(0.89,1.44) | | | |  |
| Multiple myeloma | 2 | | mix | | All | | All | | fixed | | 0.00 | 0.72 | |  | | | 1.49(0.81,2.72) | | | |  |
| Multiple myeloma | 3 | | male | | All | | All | | fixed | | 0.00 | 0.66 | |  | | | 1.07(0.82,1.40) | | | |  |
| Multiple myeloma | 4 | | mix | | Europe | | All | | fixed | | 0.00 | 0.75 | |  | | | 1.11(0.87,1.42) | | | |  |
| Leukemia | 7 | | mix | | All | | All | | fixed | | 0.00 | 0.93 | | 0.11 | | | 1.11(0.93,1.31) | | | |  |
| Leukemia | 3 | | mix | | All | | All | | fixed | | 0.00 | 0.91 | |  | | | 1.35(0.89,2.03) | | | |  |
| Leukemia | 4 | | male | | All | | All | | fixed | | 0.00 | 0.90 | |  | | | 1.06(0.88,1.28) | | | |  |
| Leukemia | 4 | | mix | | All | | Welders | | fixed | | 0.00 | 0.93 | |  | | | 1.30(0.87,1.93) | | | |  |
| Leukemia | 2 | | mix | | All | | Cement Industry Workers | | fixed | | 0.00 | 0.68 | |  | | | 1.05(0.86,1.27) | | | |  |
| **Abbreviations:** | |  | |  | |  | |  | |  | | |  | |  |  | |  |  |  |  |
|  | |  | |  | |  | |  | |  | | |  | |  |  | |  |  |  |  |

I^2^: the percentage of total variation across studies due to heterogeneity rather than chance; SIR: standardized incidence ratio; CI: confidence interval
