# Supplementary material for: The Effect of Hexavalent Chromium on the Incidence and Mortality of Human Cancers: A Meta-Analysis Based on Published Epidemiological Cohort Studies
Source: Front Oncol. 2019 Feb 4;9:24. doi: 10.3389/fonc.2019.00024 (PMC6369173; doi:10.3389/fonc.2019.00024)
Supplement: Supplementary file 3 [file Table_3.DOCX]

**The effect of hexavalent chromium on the mortality and morbidity of human cancers: A meta-analysis based on published epidemiological cohort studies**

**Authors:** Yujiao Deng1,2,†, Meng Wang1,2,†, Tian Tian1,2,†, Shuai Lin1, Peng Xu1, Linghui Zhou1, Cong Dai1, Qian Hao1, Ying Wu1, Zhen Zhai1, Yue Zhu1, Guihua Zhuang3, and Zhijun Dai1,2*

***Correspondence to:** Zhijun Dai, (E-Mail: dzj0911@126.com), or Guihua Zhuang, (E-Mail: [zhuanggh@xjtu.edu.cn](mailto:zhuanggh@xjtu.edu.cn)).

**Supplementary table 3. Studies excluded from the meta-analysis**

| **No** | **Author(s)** | **Publication year** | **Reference** | **Reason for exclusion** |  |
| --- | --- | --- | --- | --- | --- |
| 1 | Arnold M | 2018 | J Eur Acad Dermatol Venereol. 2018 Apr 28. | Not related to chrome |  |
| 2 | Levašič V | 2018 | Acta Orthop. 2018 Apr;89(2):234-239. | Not related to chrome |  |
| 3 | Burgard B | 2018 | Anticancer Res. 2018 Feb;38(2):1187-1199. | Not related to chrome |  |
| 4 | Balk SJ | 2018 | Pediatrics. 2017 Dec;140(6). pii: e20171680. | Not related to chrome |  |
| 5 | Bernthal NM | 2018 | J Surg Oncol. 2018 Mar;117(3):443-450. | Not related to chrome |  |
| 6 | Park JS | 2017 | Jpn J Clin Oncol. 2017 Nov 1;47(11):995-1001. | Not related to chrome |  |
| 7 | Sánchez-Villegas A | 2017 | Eur J Nutr. 2017 Sep 4. | Not related to chrome |  |
| 8 | Oddone E | 2017 | Am J Ind Med. 2017 Oct;60(10):852-866. doi: 10.1002/ajim.22750. | Related to asbestos |  |
| 9 | Ferrante D | 2017 | Occup Environ Med. 2017 Dec;74(12):887-898. doi: 10.1136/oemed-2016-104100. | Not related to chrome |  |
| 10 | Gupta S | 2017 | Bone Joint J. 2017 Jul;99-B(7):973-978. doi: 10.1302/0301-620X.99B7.BJJ-2016-0996. | Not related to chrome |  |
| 11 | Kauermann G | 2017 | J Expo Sci Environ Epidemiol. 2018 Jan;28(1):69-75. doi: 10.1038/jes.2017.4. | Has no available data |  |
| 12 | Vogel RI, | 2017 | Cancer Epidemiol Biomarkers Prev. 2017 Apr;26(4):607-613. | Not related to chrome |  |
| 13 | David E, | 2017 | Ann Palliat Med. 2017 Apr;6(2):118-124. | Not related to chrome |  |
| 14 | Dunlap R, | 2017 | J Invest Dermatol. 2017 Jun;137(6):1234-1239. | Not related to chrome |  |
| 15 | Ghiasvand R | 2017 | Am J Epidemiol. 2017 Feb 1;185(3):147-156. | Not related to chrome |  |
| 16 | [Janssen I.,](https://www.embase.com/search/results) | 2017 | Spine Journal 2017 17:6 (837-844) | Not related to chrome |  |
| 17 | [Guy G.P.,](https://www.embase.com/search/results) | 2017 | Journal of the American Academy of Dermatology 2017 76:2 (226-233) | Not related to chrome |  |
| 18 | Michel J, | 2016 | Int J Oral Maxillofac Surg. 2017 Apr;46(4):422-427. | Has no available data |  |
| 19 | Yang K, | 2016 | J Dermatol Sci. 2017 Mar;85(3):253-256. | Not related to chrome |  |
| 20 | McNoe BM, | 2016 | N Z Med J. 2016 Dec 2;129(1446):84-88. | Not related to chrome |  |
| 21 | Pagoto SL, | 2016 | JMIR Res Protoc. 2016 Nov 29;5(4):e228. | Not related to chrome |  |
| 22 | Smit AK, | 2016 | Cancer Epidemiol Biomarkers Prev. 2017 Feb;26(2):212-221. | Not related to chrome |  |
| 23 | Sauzet O, | 2016 | BMC Cancer. 2016 Oct 6;16(1):771. Review. | Not related to chrome |  |
| 24 | Jamshidi K, | 2016 | J Orthop Sci. 2017 Jan;22(1):69-74. | Not related to chrome |  |
| 25 | Sadetzki S, | 2016 | J Neurooncol. 2016 Dec;130(3):505-515. Epub 2016 Sep 23. | Not related to chrome |  |
| 26 | Ghiasvand R, | 2016 | J Clin Oncol. 2016 Nov 20;34(33):3976-3983. | Not related to chrome |  |
| 27 | Donato F, | 2016 | Int Arch Occup Environ Health. 2016 Nov;89(8):1155-1168. | [Meta-analysis](https://www.ncbi.nlm.nih.gov/pubmed/27604876) |  |
| 28 | Li WQ, | 2016 | Am J Public Health. 2016 Sep;106(9):1677-83. | Not related to chrome |  |
| 29 | Yang HY, | 2016 | Occup Environ Med. 2016 Aug;73(8):528-36. | Not related to chrome |  |
| 30 | Li Y | 2016 | 2016 May 24;96(19):1500-4. doi: 10.3760/cma.j.issn.0376-2491.2016.19.008. Chinese. | Not related to chrome |  |
| 31 | Guida P | 2016 | Injury. 2016 Jun;47(6):1222-8. | Not related to chrome |  |
| 32 | Liu XW, | 2016 | Eur Radiol. 2017 Jan;27(1):120-127. | Not related to chrome |  |
| 33 | Li D, | 2016 | World J Surg Oncol. 2016 Apr 19;14:114. | Not related to chrome |  |
| 34 | Foster KA, | 2016 | World Neurosurg. 2016 Nov;95:414-418. | Not related to chrome |  |
| 35 | Jawad MS, | 2016 | J Neurosurg Spine. 2016 Jun;24(6):928-36. | Not related to chrome |  |
| 36 | Murdocca M, | 2016 | Oncotarget. 2016 Mar 22;7(12):14765-80. | Not related to chrome |  |
| 37 | Lu B, | 2016 | Am J Public Health. 2016 Apr;106(4):698-706. | Not related to chrome |  |
| 38 | Rosario M, | 2016 | Am J Public Health. 2016 Apr;106(4):698-706. | Has no available data |  |
| 39 | [Lacourt A.,](https://www.embase.com/search/results) | 2016 | European Respiratory Journal 2016 48 Supplement 60 | Not related to chrome |  |
| 40 | Panou V. | 2015 | Journal of Thoracic Oncology 2015 10:9 SUPPL. 2 (S348-) | Not related to chrome |  |
| 41 | Welling R. | 2015 | Occupational and Environmental Medicine 2015 72:2 (151-159) | Meta-analysis |  |
| 42 | Parkins G. | 2015 | British Journal of Dermatology 2014 171 SUPPL. 4 (20-) | Not related to chrome |  |
| 43 | Girardi P | 2015 | Epidemiol Prev. 2015;39(3):183-7. Italian. | Has no available data |  |
| 44 | Lee SH, | 2015 | J Neurooncol. 2016 Feb;126(3):509-17. doi: 10.1007/s11060-015-1990-z. Epub 2015 Dec 7. | Not related to chrome |  |
| 45 | Shigita K, | 2015 | J Gastroenterol Hepatol. 2016 May;31(5):973-9. | Not related to chrome |  |
| 46 | Toğral G, | 2015 | Acta Orthop Traumatol Turc. 2015;49(6):634-40. | Not related to chrome |  |
| 47 | Levy-Shraga Y, | 2015 | PLoS One. 2015 Sep 8;10(9):e0137453. | Not related to chrome |  |
| 48 | Baitar A, | 2015 | J Geriatr Oncol. 2015 Sep;6(5):401-10. | Not related to chrome |  |
| 49 | Moussazadeh N, | 2015 | Int J Radiat Oncol Biol Phys. 2015 Oct 1;93(2):361-7. | Not related to chrome |  |
| 50 | Dabravolski D, | 2015 | Orthopade. 2015 Oct;44(10):806-19. | Not related to chrome |  |
| 51 | Farley C, | 2015 | J Surg Oncol. 2015 Aug;112(2):183-7. | Not related to chrome |  |
| 52 | Novikov SM, | 2015 | Gig Sanit. 2015 Mar-Apr;94(2):88-92. Russian. | Not related to chrome |  |
| 53 | Boeke CE, | 2015 | Cancer Causes Control. 2015 Aug;26(8):1181-7. | Not related to chrome |  |
| 54 | Rajah G, | 2015 | J Neurosurg Spine. 2015 Aug;23(2):228-32. | Not related to chrome |  |
| 55 | Binazzi A, | 2015 | BMC Cancer. 2015 Feb 13;15:49. | [A review and meta-analysis.](https://www.ncbi.nlm.nih.gov/pubmed/25885319) | |
| 56 | Molinaro AM, | 2015 | Am J Epidemiol. 2015 Jun 1;181(11):908-16. | Not related to chrome |  |
| 57 | Danjou AM, | 2015 | Breast Cancer Res. 2015 Mar 17;17:39. | Not related to chrome |  |
| 58 | Bernthal NM, | 2015 | Ann Surg Oncol. 2015 Nov;22(12):3921-8. | Not related to chrome |  |
| 59 | Peluso ME, | 2015 | Mutagenesis. 2015 Jul;30(4):519-25. | Has no available data |  |
| 60 | Zhu Y | 2015 | Oncol Lett. 2015 Mar;9(3):1073-1080. | Not related to chrome |  |
| 61 | Patrascu JM, | 2015 | Eur Rev Med Pharmacol Sci. 2014;18(24):3898-901. | Not related to chrome |  |
| 62 | Samper Wamba JD, | 2015 | Radiologia. 2015 Sep-Oct;57(5):402-11. | Not related to chrome |  |
| 63 | Rajani R, | 2015 | J Foot Ankle Surg. 2015 Nov-Dec;54(6):1141-5. | Not related to chrome |  |
| 64 | Keçeci B, | 2014 | Acta Orthop Traumatol Turc. 2014;48(5):500-6. | Not related to chrome |  |
| 65 | Wong CC, | 2014 | Australas J Dermatol. 2015 Nov;56(4):290-3. | Has no available data |  |
| 66 | Koh DH, | 2014 | Occup Med (Lond). 2015 Jan;65(1):72-7. | Has no available data |  |
| 67 | Ortega-Guerrero MA, | 2014 | Occup Environ Med. 2015 Mar;72(3):216-8. | Has no available data |  |
| 68 | Hill T, | 2014 | ANZ J Surg. 2015 Mar;85(3):159-63. | Not related to chrome |  |
| 69 | Rao PJ, | 2014 | Orthop Surg. 2014 Aug;6(3):187-95. | Not related to chrome |  |
| 70 | Cohen SS, | 2014 | Occup Environ Med. 2014 Nov;71(11):796-802. | [A review and meta-analysis](https://www.ncbi.nlm.nih.gov/pubmed/25143515) |  |
| 71 | Blashill AJ, | 2014 | Am J Public Health. 2014 Sep;104(9):1640-1. | Not related to chrome |  |
| 72 | Bagheri R, | 2014 | J Cardiothorac Surg. 2014 Jun 19;9:106. | Not related to chrome |  |
| 73 | Bonilla C, | 2014 | BMC Public Health. 2014 Jun 12;14:597. | Not related to chrome |  |
| 74 | Luqman M, | 2014 | Asian Pac J Cancer Prev. 2014;15(7):3035-9. | Has no available data |  |
| 75 | Li Z, | 2014 | Chin Med J (Engl). 2014;127(8):1493-6. | Not related to chrome |  |
| 76 | Roberts AL, | 2014 | J Adolesc Health. 2014 Sep;55(3):373-9. | Not related to chrome |  |
| 77 | Xing R, | 2014 | Acta Orthop Belg. 2013 Dec;79(6):731-7. | Not related to chrome |  |
| 78 | Oddone E, | 2014 | Med Lav. 2014 Jan-Feb;105(1):15-29. Italian. | Related to asbestos |  |
| 79 | Gerosa A, | 2014 | Epidemiol Prev. 2013 Nov-Dec;37(6):376-85. Italian. | Has no available data |  |
| 80 | Reddy KI, | 2014 | Clin Orthop Relat Res. 2014 Jun;472(6):1901-10. | Not related to chrome |  |
| 81 | Veierød MB, | 2014 | Int J Cancer. 2014 Jul 15;135(2):413-22. | Not related to chrome |  |
| 82 | Haney JT Jr, | 2014 | Regul Toxicol Pharmacol. 2014 Mar;68(2):201-11. | Has no available data |  |
| 83 | Corcos G, | 2014 | Spine (Phila Pa 1976). 2014 Mar 1;39(5):E332-8. | Not related to chrome |  |
| 84 | Lee, Y.-H., et al. | 2014 | PLoS One 9(9). | Not related to chrome |  |
| 85 | Ahn Y.-S. | 2014 | Occupational and Environmental Medicine 2014 71 SUPPL. 1 (A44-A45) | Not related to chrome |  |
| 86 | Ahn Y.S. | 2013 | Occupational and Environmental Medicine 2013 70 SUPPL. 1 | Not related to chrome |  |
| 87 | Zhang M. | 2013 | Journal of Investigative Dermatology 2013 133 SUPPL. 1 (S96-) | Not related to chrome |  |
| 88 | Akasaka J, | 2013 | Int J Gynecol Cancer. 2013 Oct;23(8):1501-5. | Not related to chrome |  |
| 89 | Minatel E, | 2013 | Lung Cancer. 2014 Jan;83(1):78-82. | Not related to chrome |  |
| 90 | Campanacci DA, | 2013 | Injury. 2014 Feb;45(2):399-404. | Not related to chrome |  |
| 91 | Barzilay Y, | 2013 | Spine (Phila Pa 1976). 2014 Jan 15;39(2):153-7. | Not related to chrome |  |
| 92 | Wong AP, | 2013 | J Neurooncol. 2013 Dec;115(3):493-503. | Not related to chrome |  |
| 93 | Wei Y, | 2013 | PLoS One. 2013 Oct 15;8(10):e77413. | Has no available data |  |
| 94 | Zhang M, | 2013 | Cancer Epidemiol Biomarkers Prev. 2013 Dec;22(12):2425-9. | Not related to chrome |  |
| 95 | Piccioli A, | 2013 | Injury. 2014 Feb;45(2):412-7. | Not related to chrome |  |
| 96 | Järvholm B, | 2013 | Am J Ind Med. 2014 Jan;57(1):49-55. | Related to asbestos |  |
| 97 | Choi MG, | 2013 | Gastric Cancer. 2014;17(3):556-61. | Not related to chrome |  |
| 98 | Couraud S, | 2013 | Rev Mal Respir. 2013 Sep;30(7):576-83. | Not related to chrome |  |
| 99 | Zhivin S, | 2013 | Am J Ind Med. 2013 Nov;56(11):1262-71. | Not related to chrome |  |
| 100 | Khalesi M, | 2013 | Cancer Epidemiol. 2013 Oct;37(5):534-43. | Not related to chrome |  |
| 101 | Wallace MT, | 2013 | J Pediatr Orthop. 2014 Jan;34(1):92-100. | Not related to chrome |  |
| 102 | Price SL, | 2013 | Clin Orthop Relat Res. 2013 Oct;471(10):3303-7. | Not related to chrome |  |
| 103 | Subramanian S, | 2013 | JAMA Otolaryngol Head Neck Surg. 2013 May;139(5):489-95. | Not related to chrome |  |
| 104 | Van Humbeeck L, | 2013 | Palliat Med. 2013 Jul;27(7):583-95. | Not related to chrome |  |
| 105 | Saberi Hosnijeh F, | 2013 | Occup Environ Med. 2013 Jul;70(7):464-70. | Not related to chrome |  |
| 106 | Rosenberg AR, | 2013 | JAMA Pediatr. 2013 Jun;167(6):537-43. | Not related to chrome |  |
| 107 | Wu WT, | 2013 | Am J Ind Med. 2013 Jun;56(6):701-8. | Has no available data |  |
| 108 | Bancone C, | 2012 | G Ital Med Lav Ergon. 2012 Jul-Sep;34(3 Suppl):492-4. Italian. | Has no available data |  |
| 109 | Manjila S, | 2012 | J Neurosurg. 2013 Jul;119(1):113-20. | Not related to chrome |  |
| 110 | Quraishi NA, | 2013 | Eur Spine J. 2013 Mar;22 Suppl 1:S27-32. | Not related to chrome |  |
| 111 | Nakamura T, | 2013 | Int Orthop. 2013 Apr;37(4):647-51. | Not related to chrome |  |
| 112 | Hara T, | 2012 | J UOEH. 2012 Dec 1;34(4):309-13. Japanese. | Has no available data |  |
| 113 | Tóth V, | 2012 | Pathol Oncol Res. 2013 Apr;19(2):323-8. | Not related to chrome |  |
| 114 | Koh DH, | 2012 | Am J Ind Med. 2013 Mar;56(3):276-81. | Duplicated data |  |
| 115 | Crane LA, | 2012 | Am J Prev Med. 2012 Oct;43(4):399-410. | Not related to chrome |  |
| 116 | Schnatter A.R. | 2012 | Occupational and Environmental Medicine 2012 69:12 (877-882) | Not related to chrome |  |
| 117 | Olsen C.M. | 2012 | Not related to chrome | Not related to chrome |  |
| 118 | Zhang M. | 2011 | Cancer Prevention Research 2011 4:10 SUPPL. 1 | Not related to chrome |  |
| 119 | Olsen CM, | 2012 | Int J Epidemiol. 2012 Aug;41(4):929-929i. | Not related to chrome |  |
| 120 | Kenborg L, | 2012 | Mov Disord. 2012 Sep 1;27(10):1283-9. | Not related to CANCER |  |
| 121 | Rachiotis G, | 2012 | BMC Pulm Med. 2012 Jun 27;12:30. | Has no available data |  |
| 122 | Kuijpens JH, | 2012 | Eur J Cancer. 2012 Oct;48(15):2369-74. | Not related to chrome |  |
| 123 | La Maida GA, | 2012 | Eur Spine J. 2012 May;21 Suppl 1:S61-8. | Not related to chrome |  |
| 124 | Zhang M, | 2012 | J Clin Oncol. 2012 May 10;30(14):1588-93. | Not related to chrome |  |
| 125 | Lostritto K, | 2012 | BMC Public Health. 2012 Feb 10;12:118. | Not related to chrome |  |
| 126 | La Vecchia C, | 2012 | Eur J Cancer Prev. 2012 May;21(3):227-30. | Related to asbestos |  |
| 127 | Trumm CG, | 2012 | Skeletal Radiol. 2012 Nov;41(11):1391-400. | Not related to chrome |  |
| 128 | Ye SM, | 2012 | Zhongguo Gu Shang. 2011 Dec;24(12):977-81. Chinese. | Not related to chrome |  |
| 129 | Wei-Passanese EX, | 2012 | Photochem Photobiol. 2012 Mar-Apr;88(2):483-9. | Not related to chrome |  |
| 130 | Yang Z, | 2012 | Eur Spine J. 2012 May;21(5):912-9. | Not related to chrome |  |
| 131 | Menegozzo S, | 2011 | Ann Ist Super Sanita. 2011;47(3):296-304. | Related to asbestos |  |
| 132 | Eyesan SU, | 2011 | Niger J Clin Pract. 2011 Apr-Jun;14(2):146-50. | Not related to chrome |  |
| 133 | Giordano F, | 2011 | Int Arch Occup Environ Health. 2012 May;85(4):373-9. | Only SMRs, no CIs |  |
| 134 | Boschi V, | 2011 | Scand J Surg. 2011;100(2):120-4. | Not related to chrome |  |
| 135 | Salerno C, | 2011 | Ann Ig. 2011 Jan-Feb;23(1):33-42. Italian. | Has no available data |  |
| 136 | Lin WH, | 2011 | Clin Orthop Relat Res. 2011 Nov;469(11):3200-8. | Not related to chrome |  |
| 137 | Bakos RM, | 2011 | Eur J Dermatol. 2011 Sep-Oct;21(5):705-9. | Not related to chrome |  |
| 138 | Brouse CH, | 2011 | Prev Chronic Dis. 2011 Jul;8(4):A88. Epub 2011 Jun 15. | Not related to chrome |  |
| 139 | Akamatsu N, | 2011 | J Gastroenterol Hepatol. 2011 Dec;26(12):1795-803. | Not related to chrome |  |
| 140 | Dessinioti C, | 2011 | Exp Dermatol. 2011 Aug;20(8):622-6. | Not related to chrome |  |
| 141 | Shousha M, | 2011 | Spine (Phila Pa 1976). 2012 Jan 1;37(1):E30-6. | Not related to chrome |  |
| 142 | Butt FM, | 2011 | J Craniomaxillofac Surg. 2012 Feb;40(2):e39-45. | Not related to chrome |  |
| 143 | Berwick M. | 2011 | Cancer Epidemiol Biomarkers Prev. 2011 Apr;20(4):582-4. | Not related to chrome |  |
| 144 | Redal-Baigorri B, | 2011 | Dan Med Bull. 2011 Feb;58(2):A4236. | Not related to chrome |  |
| 145 | Karlsson MA, | 2011 | Br J Dermatol. 2011 Apr;164(4):830-7. | Not related to chrome |  |
| 146 | Baker MK, | 2010 | Arch Dermatol. 2010 Dec;146(12):1427-8. | Not related to chrome |  |
| 147 | Choi K, | 2010 | Arch Dermatol. 2010 Dec;146(12):1356-61. | Not related to chrome |  |
| 148 | Inoue M, | 2010 | J Epidemiol Community Health. 2012 May;66(5):448-56. | Not related to chrome |  |
| 149 | Nguyen MH, | 2010 | Biol Blood Marrow Transplant. 2011 Jul;17(7):1043-50. | Not related to chrome |  |
| 150 | Jensen JD, | 2010 | Clin Dermatol. 2010 Nov-Dec;28(6):644-9. | Not related to chrome |  |
| 151 | Mislowsky A, | 2010 | Ann Surg Oncol. 2011 Mar;18(3):745-51. | Not related to chrome |  |
| 152 | Neasham D, | 2010 | Occup Environ Med. 2011 Jan;68(1):77-81. | Not related to chrome |  |
| 153 | Barbieri PG, | 2010 | G Ital Med Lav Ergon. 2010 Apr-Jun;32(2):149-53. Italian. | Related to asbestos |  |
| 154 | Finkelstein MM. | 2010 | Am J Ind Med. 2010 Nov;53(11):1065-9. | Related to asbestos |  |
| 155 | Ibfelt E, | 2010 | Occup Environ Med. 2010 Nov;67(11):772-7. | Not related to cancer |  |
| 156 | Yang XR, | 2010 | Fam Cancer. 2010 Dec;9(4):625-33. | Not related to chrome |  |
| 157 | Agarwal M, | 2010 | Clin Orthop Relat Res. 2010 Nov;468(11):2904-13. | Not related to chrome |  |
| 158 | Veierød MB, | 2010 | Cancer Epidemiol Biomarkers Prev. 2010 Jun;19(6):1569-76. | Not related to chrome |  |
| 159 | de Camargo OP, | 2010 | Clin Orthop Relat Res. 2010 Nov;468(11):2969-75. | Not related to chrome |  |
| 160 | Mosher CE, | 2010 | Arch Dermatol. 2010 Apr;146(4):412-7. | Not related to chrome |  |
| 161 | Huang W, | 2010 | Spine (Phila Pa 1976). 2010 Apr 15;35(8):E278-84. | Not related to chrome |  |
| 162 | Hong SK, | 2010 | World J Urol. 2010 Dec;28(6):721-6. | Not related to chrome |  |
| 163 | Aalborg J, | 2010 | Arch Dermatol. 2009 Sep;145(9):989-96. | Not related to chrome |  |
| 164 | Kumagai S, | 2009 | Am J Ind Med. 2009 Oct;52(10):790-8. | Not related to chrome |  |
| 165 | Eyesan SU, | 2009 | Niger J Clin Pract. 2009 Dec;12(4):367-70. | Not related to chrome |  |
| 166 | Chang CS, | 2009 | J Plast Reconstr Aesthet Surg. 2010 Aug;63(8):1265-8. | Not related to chrome |  |
| 167 | Noar MD. | 2009 | Gastrointest Endosc. 2009 Sep;70(3):454-6. | Not related to chrome |  |
| 168 | Menendez LR, | 2009 | Clin Orthop Relat Res. 2009 Nov;467(11):2831-7. | Not related to chrome |  |
| 169 | Jeyarajah S, | 2009 | Colorectal Dis. 2011 Jan;13(1):31-8. | Has no available data |  |
| 170 | Pronk A, | 2009 | Occup Environ Med. 2009 Oct;66(10):672-8. | Not related to chrome |  |
| 171 | Cable C, | 2009 | Bone Marrow Transplant. 2010 Jan;45(1):31-7. | Not related to chrome |  |
| 172 | Tarrés J, | 2009 | Arch Bronconeumol. 2009 Sep;45(9):429-34. | Related to asbestos |  |
| 173 | Krokidis M, | 2009 | Cardiovasc Intervent Radiol. 2010 Feb;33(1):97-106. | Not related to chrome |  |
| 174 | Loew BJ, | 2009 | Gastrointest Endosc. 2009 Sep;70(3):445-53. | Not related to chrome |  |
| 175 | Mourali M, | 2009 | Tunis Med. 2008 Jul;86(7):665-9. | Not related to chrome |  |
| 176 | Georgy BA. | 2009 | AJNR Am J Neuroradiol. 2009 Jun;30(6):1197-202. | Not related to chrome |  |
| 177 | Barbieri PG, | 2009 | G Ital Med Lav Ergon. 2008 Oct-Dec;30(4):329-33. Italian. | Related to asbestos |  |
| 178 | McArthur N, | 2009 | Orthopedics. 2009 Feb;32(2):90. | Not related to chrome |  |
| 179 | Roberts DJ, | 2009 | Clin Pediatr (Phila). 2009 Jul;48(6):614-22. | Not related to chrome |  |
| 180 | Sun TD, | 2009 | Zhonghua Lao Dong Wei Sheng Zhi Ye Bing Za Zhi. 2008 Oct;26(10):605-8. Chinese. | [Exposed to asbestos: a meta-analysis](https://www.ncbi.nlm.nih.gov/pubmed/19272256) | |
| 181 | Bertolotti M, | 2009 | Epidemiol Prev. 2008 Jul-Oct;32(4-5):218-28. Italian. | Related to asbestos |  |
| 182 | Siew SS, | 2009 | Scand J Work Environ Health. 2008 Dec;34(6):444-50. | Just RR, no SIR or SMR and CIs |  |
| 183 | Niu XH, | 2009 | Zhonghua Wai Ke Za Zhi. 2008 Nov 15;46(22):1730-3. Chinese. | Not related to chrome |  |
| 184 | Tseng YY, | 2009 | Minim Invasive Neurosurg. 2008 Oct;51(5):280-4. | Not related to chrome |  |
| 185 | Sichletidis L, | 2009 | Respiration. 2009;78(1):63-8. | Has no available data |  |
| 186 | Su CC, | 2009 | Oral Oncol. 2008 Nov;44(11):1032-8. | Not related to chrome |  |
| 187 | Gotoda T, | 2009 | Gastrointest Endosc. 2009 Jan;69(1):10-5. | Not related to chrome |  |
| 188 | Barone-Adesi F, | 2009 | Int J Cancer. 2008 Aug 15;123(4):912-6. | Related to asbestos |  |
| 189 | Dasgupta S, | 2009 | J Postgrad Med. 2008 Apr-Jun;54(2):115-25. Review. | Not related to cancer, review |  |
| 190 | Nagornaia AM, | 2009 | Med Tr Prom Ekol. 2008;(3):27-33. Russian. | Related to asbestos |  |
| 191 | Moshammer H, | 2009 | Int Arch Occup Environ Health. 2009 Jan;82(2):199-207. | Related to asbestos |  |
| 192 | Anselmetti GC, | 2008 | Cardiovasc Intervent Radiol. 2008 Sep-Oct;31(5):937-47. | Not related to cancer |  |
| 193 | Lope V, | 2008 | Int Arch Occup Environ Health. 2009 Jan;82(2):267-74. | Just RR, no SIR or SMR and CIs |  |
| 194 | Audisio R, | 2008 | Med Lav. 2008 Jan-Feb;99(1):40-8. Italian. | Has no available data |  |
| 195 | Adaş M, | 2007 | Acta Orthop Traumatol Turc. 2007 Nov-Dec;41(5):380-6. Turkish. | Not related to chrome |  |
| 196 | Ting W, | 2007 | Int J Dermatol. 2007 Dec;46(12):1253-7. | Not related to chrome |  |
| 197 | Ferrante D, | 2007 | Environ Health Perspect. 2007 Oct;115(10):1401-5. | Related to asbestos |  |
| 198 | Yiin JH, | 2007 | Radiat Res. 2007 Sep;168(3):341-8. | Not related to chrome |  |
| 199 | Magnani C, | 2007 | Occup Environ Med. 2008 Mar;65(3):164-70. Epub 2007 Aug 17. | Related to asbestos |  |
| 200 | Baird CJ, | 2007 | J Neurosurg. 2007 Aug;107(2):347-51. | Not related to chrome |  |
| 201 | Battista G, | 2007 | Med Lav. 2007 Jul-Aug;98(4):289-95. Italian. | Not related to chrome |  |
| 202 | Sacco A, | 2007 | Med Lav. 2007 May-Jun;98(3):252-4. | Related to asbestos |  |
| 203 | Bonneterre V, | 2007 | Occup Med (Lond). 2007 Sep;57(6):438-43. Epub 2007 Jun 24. Review. | Has no available data |  |
| 204 | Boston SE, | 2007 | Vet Surg. 2007 Jun;36(4):314-23. | Not related to chrome |  |
| 205 | Marsh GM, | 2007 | Regul Toxicol Pharmacol. 2007 Aug;48(3):308-19. Epub 2007 Apr 30. | Has no available data |  |
| 206 | Chacko AG, | 2007 | J Clin Neurosci. 2007 Aug;14(8):764-9. Epub 2007 May 25. | Not related to chrome |  |
| 207 | Everatt RP, | 2007 | Am J Ind Med. 2007 Jun;50(6):455-63. | Related to asbestos |  |
| 208 | Raffaelli I, | 2007 | Med Lav. 2007 Mar-Apr;98(2):156-63. Italian. | Related to asbestos |  |
| 209 | Dodic Fikfak M, | 2007 | Ann Occup Hyg. 2007 Apr;51(3):261-8. Epub 2007 Mar 9. | Related to asbestos, case-control study | |
| 210 | Baltayiannis N, | 2007 | J BUON. 2006 Oct-Dec;11(4):457-62. | Not related to chrome |  |
| 211 | Pritsch T, | 2007 | Clin Orthop Relat Res. 2007 May;458:159-67. | Not related to chrome |  |
| 212 | Sjödahl K, | 2007 | nt J Cancer. 2007 May 1;120(9):2013-8. | Just IRR, no SIR or SMR and CIs |  |
| 213 | Roberti S, | 2006 | Epidemiol Prev. 2006 Jul-Oct;30(4-5):232-6. Italian. | Not related to chrome |  |
| 214 | Fano V, | 2006 | Epidemiol Prev. 2006 Jul-Oct;30(4-5):221-6. Italian. | Not related to chrome |  |
| 215 | Yang Z, | 2006 | Zhongguo Xiu Fu Chong Jian Wai Ke Za Zhi. 2006 Oct;20(10):999-1003. Chinese. | Not related to chrome |  |
| 216 | [The International Agency](https://onlinelibrary.wiley.com/action/doSearch?ContribAuthorStored=The+International+Agency+For+Research+On+Cancer+Working+Group+On+Artificial+Ultraviolet+UV+Light+And+Skin+Cancer) | 2007 | Int J Cancer. 2007 Mar 1;120(5):1116-22. Review. | Not related to chrome |  |
| 217 | Meguellati-Hakkas D, | 2006 | J Occup Environ Med. 2006 Nov;48(11):1166-72. | Related to asbestos |  |
| 218 | Rahu K, | 2006 | Ann Epidemiol. 2006 Dec;16(12):917-9. Epub 2006 Oct 5. | Not related to cancer |  |
| 219 | Tuchinda C, | 2006 | Photodermatol Photoimmunol Photomed. 2006 Oct;22(5):247-53. | Not related to chrome |  |
| 220 | Purdue MP, | 2006 | Scand J Work Environ Health. 2006 Aug;32(4):270-5. | Just RR, no SIR or SMR and CIs |  |
| 221 | Ji J, Hemminki K. | 2006 | Eur J Cancer Prev. 2006 Oct;15(5):391-7. | Has no available data |  |
| 222 | DeChello LM, | 2006 | Int J Health Geogr. 2006 Aug 2;5:31. | Not related to cancer |  |
| 223 | Fevotte J, | 2006 | Ann Occup Hyg. 2006 Nov;50(8):765-75. Epub 2006 Jul 13. | Has no available data, case-control study | |
| 224 | Veyalkin I, | 2006 | Ind Health. 2006 Jan;44(1):69-74. | PMRs |  |
| 225 | Ramos L, | 2006 | Eur J Haematol. 2006 Jul;77(1):7-13. Epub 2006 Apr 11. | Not related to cancer |  |
| 226 | Michaels D, | 2006 | Environ Health. 2006 Feb 23;5:5. Review. | Not related to cancer |  |
| 227 | Haraguchi S, | 2006 | Surg Today. 2006;36(3):225-9. | Not related to chrome |  |
| 228 | Barragán-Campos HM, | 2006 | Radiology. 2006 Jan;238(1):354-62. | Not related to chrome |  |
| 229 | Fedi A, Blagini B, | 2005 | Med Lav. 2005 May-Jun;96(3):243-9. Italian. | Related to asbestos |  |
| 230 | De Roos AJ, | 2005 | Ann Epidemiol. 2005 Nov;15(10):762-70. | Not related to chrome |  |
| 231 | Ji J, | 2006 | Ann Epidemiol. 2006 May;16(5):370-6. Epub 2005 Oct 24. | Not related to chrome |  |
| 232 | Ji J, | 2005 | World J Urol. 2005 Sep;23(4):271-8. Epub 2005 Nov 8. | Has no available data |  |
| 233 | Mont'Alverne F, | 2005 | AJNR Am J Neuroradiol. 2005 Aug;26(7):1641-5. | Not related to chrome |  |
| 234 | Jansson C, | 2005 | Cancer Causes Control. 2005 Aug;16(6):755-64. | Has no available data |  |
| 235 | Kjaerheim K, | 2005 | Cancer Causes Control. 2005 Jun;16(5):593-8. | Related to asbestos |  |
| 236 | Yiin JH, | 2005 | Radiat Res. 2005 Jun;163(6):603-13. | Not related to chrome |  |
| 237 | Håkansson N, | 2005 | Occup Environ Med. 2005 May;62(5):304-8. | Has no available data, case-control study | |
| 238 | Luippold RS, | 2005 | J Occup Environ Med. 2005 Apr;47(4):381-5. | Has no available data |  |
| 239 | Browne ML, | 2005 | Environ Res. 2005 Jun;98(2):224-32. | Related to asbestos |  |
| 240 | Li L, | 2004 | Biomed Environ Sci. 2004 Dec;17(4):459-68. | Related to asbestos,meta-analysis | |
| 241 | Luberto F, | 2004 | Epidemiol Prev. 2004 Jul-Oct;28(4-5):239-46. Italian. | Related to asbestos |  |
| 242 | Proctor DM, | 2004 | J Occup Environ Hyg. 2004 Nov;1(11):752-67. | Has no available data |  |
| 243 | Stryker JE, | 2004 | J Adolesc Health. 2004 Dec;35(6):528.e1-9. | Not related to chrome |  |
| 244 | Park RM, | 2004 | Risk Anal. 2004 Oct;24(5):1099-108. | Not related to chrome |  |
| 245 | DeGroot H, | 2004 | Clin Orthop Relat Res. 2004 Oct;(427):190-7. | Not related to chrome |  |
| 246 | Autier P. | 2004 | Eur J Cancer. 2004 Nov;40(16):2367-76. | Not related to chrome |  |
| 247 | Meo SA. | 2004 | Saudi Med J. 2004 Sep;25(9):1153-9. Review. | Review |  |
| 248 | Harrison RM, | 2004 | Occup Environ Med. 2004 Oct;61(10):799-805. | Not related to chrome |  |
| 249 | Lindholm C, | 2004 | Cancer. 2004 Nov 1;101(9):2067-78. | Not related to chrome |  |
| 250 | Liptak JM, | 2004 | Vet Surg. 2004 Sep-Oct;33(5):446-56. | Not related to chrome |  |
| 251 | Monti M, | 2004 | Support Care Cancer. 2004 Nov;12(11):752-7. | Not related to chrome |  |
| 252 | Finkelstein MM, | 2004 | Occup Environ Med. 2004 Sep;61(9):736-42. | Not related to chrome |  |
| 253 | Musk AW, | 2004 | Lung Cancer. 2004 Aug;45 Suppl 1:S21-3. Review. | Review |  |
| 254 | Poetker DM, | 2004 | Otol Neurotol. 2004 Jul;25(4):604-9. | Not related to chrome |  |
| 255 | Smailyte G, | 2004 | Occup Environ Med. 2004 Jun;61(6):529-34. | Not related to chrome |  |
| 256 | Smailyte G, | 2004 | Scand J Work Environ Health. 2004 Feb;30(1):64-70. | Related to asbestos |  |
| 257 | Li L, | 2004 | Zhonghua Yu Fang Yi Xue Za Zhi. 2004 Jan;38(1):39-42. Chinese. | Not related to chrome |  |
| 258 | Kveton JF, | 2004 | Laryngoscope. 2004 Jan;114(1):33-7. | Not related to cancer |  |
| 259 | van Wijngaarden E | 2004 | Nonlinearity Biol Toxicol Med. 2004 Jan;2(1):27-34. | Review |  |
| 260 | Brown, S. C., et al. | 2004 | Am J Epidemiol 160(2): 163-172. | Not related to chrome |  |
| 261 | Tunn PU, | 2003 | Z Orthop Ihre Grenzgeb. 2003 Nov-Dec;141(6):690-8. German. | Not related to chrome |  |
| 262 | Crump C, | 2003 | Risk Anal. 2003 Dec;23(6):1147-63. | Has no available data |  |
| 263 | Geerdink EA, | 2003 | Eur J Endocrinol. 2003 Dec;149(6):577-82. | PMR |  |
| 264 | Veyalkin IV, | 2003 | Am J Ind Med. 2003 Dec;44(6):637-42. | Not related to chrome |  |
| 265 | Kauppinen T, | 2003 | Am J Ind Med. 2003 Oct;44(4):343-50. | Not related to chrome |  |
| 266 | Lee WJ, | 2003 | Int J Cancer. 2003 Oct 20;107(1):134-8. | RR |  |
| 267 | Knudsen LE, | 2003 | Ugeskr Laeger. 2003 Jul 14;165(29):2882-6. Danish. | Not related to cancer |  |
| 268 | Hemminki K, | 2003 | Arch Dermatol. 2003 Jul;139(7):885-9. | Not related to chrome |  |
| 269 | Cogle CR, | 2003 | Am J Hematol. 2003 Jul;73(3):169-75. | Not related to chrome |  |
| 270 | Cai J, | 2003 | Chin Med J (Engl). 2003 Feb;116(2):187-90. | Not related to chrome |  |
| 271 | Neuberger M, | 2003 | Int Arch Occup Environ Health. 2003 Mar;76(2):161-6. Epub 2002 Nov 20. | Related to asbestos |  |
| 272 | Satcher RL Jr, | 2003 | Clin Orthop Relat Res. 2003 Apr;(409):209-17. | Not related to chrome |  |
| 273 | MacLennan R, | 2003 | J Am Acad Dermatol. 2003 Mar;48(3):367-75. | Not related to chrome |  |
| 274 | Piatek S, | 2003 | Zentralbl Chir. 2003 Feb;128(2):131-8. German. | Not related to cancer |  |
| 275 | Zumberg MS, | 2002 | Cytotherapy. 2002;4(6):531-8. | Not related to cancer |  |
| 276 | Ulvestad B, | 2002 | Scand J Work Environ Health. 2002 Dec;28(6):411-7. | Related to asbestos |  |
| 277 | Mithoefer AB, | 2002 | Liver Transpl. 2002 Oct;8(10):939-44. | Not related to chrome |  |
| 278 | Allen JW, | 2002 | Surg Endosc. 2002 Dec;16(12):1802-5. Epub 2002 Jul 29. | Not related to chrome |  |
| 279 | Coviello V, | 2002 | Epidemiol Prev. 2002 Mar-Apr;26(2):65-70. Italian. | Related to asbestos |  |
| 280 | Håkansson N, | 2002 | Occup Environ Med. 2002 Jul;59(7):481-6. | RR |  |
| 281 | Iaia TE, | 2002 | Med Lav. 2002 Mar-Apr;93(2):95-107. Italian. | Has no available data |  |
| 282 | Boyd AS, | 2002 | J Am Acad Dermatol. 2002 May;46(5):706-9. | Not related to chrome |  |
| 283 | Donati D, | 2002 | Arch Orthop Trauma Surg. 2002 Mar;122(2):123-8. | Not related to chrome |  |
| 284 | Shibahara T, | 2002 | J Oral Maxillofac Surg. 2002 Feb;60(2):182-5. | Not related to chrome |  |
| 285 | Pollán M, | 2001 | Ann Epidemiol. 2001 Nov;11(8):554-62. | Has no available data |  |
| 286 | Puntoni R, | 2001 | Am J Ind Med. 2001 Oct;40(4):363-70. | Related to asbestos |  |
| 287 | Dutka J, | 2000 | Chir Narzadow Ruchu Ortop Pol. 2000;65(6):643-9. Polish. | Not related to chrome |  |
| 288 | Szadkowska-Stańczyk I, | 2001 | Am J Ind Med. 2001 Jun;39(6):547-56. | Not related to chrome |  |
| 289 | Christman NJ, | 2001 | Oncol Nurs Forum. 2001 Jan-Feb;28(1):93-8. | Not related to chrome |  |
| 290 | Sorger JI, | 2001 | Clin Orthop Relat Res. 2001 Jan;(382):66-74. | Not related to chrome |  |
| 291 | Mándi A, | 2000 | Int Arch Occup Environ Health. 2000 Nov;73(8):555-60. | Related to asbestos |  |
| 292 | Tanigawa N, | 2000 | Acta Radiol. 2000 Sep;41(5):425-8. | Not related to chrome |  |
| 293 | Szeszenia-Dabrowska N, | 2000 | Int J Occup Med Environ Health. 2000;13(2):121-30. | Related to asbestos |  |
| 294 | Friedman CD, | 2000 | Arch Facial Plast Surg. 2000 Apr-Jun;2(2):124-9. | Not related to cancer |  |
| 295 | Gibb HJ, | 2000 | Am J Ind Med. 2000 Aug;38(2):115-26. | Has no available data |  |
| 296 | Jalal A, | 2000 | Eur J Cardiothorac Surg. 2000 Apr;17(4):370-6. Review. | Has no available data, review |  |
| 297 | Danielsen TE, | 2000 | J Occup Environ Med. 2000 Jan;42(1):101-9. | Has no available data |  |
| 298 | Case BW, | 2000 | Inhal Toxicol. 2000 Jan;12 Suppl 3:411-8. | Related to asbestos |  |
| 299 | Chakravarti A, | 1999 | J Bone Joint Surg Am. 1999 Nov;81(11):1566-73. | Not related to chrome |  |
| 300 | Klotch DW, | 1999 | Otolaryngol Head Neck Surg. 1999 Oct;121(4):388-92. | Not related to chrome |  |
| 301 | Blackley HR, | 1999 | J Bone Joint Surg Am. 1999 Jun;81(6):811-20. | Not related to chrome |  |
| 302 | de Silva SR, | 1999 | J Occup Environ Med. 1999 Jun;41(6):464-8. | Not related to chrome |  |
| 303 | Malerba M, | 1999 | World J Surg. 1999 Jul;23(7):670-5. | Not related to chrome |  |
| 304 | Germani D, | 1999 | Am J Ind Med. 1999 Jul;36(1):129-34. | Not related to chrome |  |
| 305 | Becker N. | 1999 | J Occup Environ Med. 1999 Apr;41(4):294-303. | Has no available data |  |
| 306 | Tulchinsky TH, | 1999 | Am J Ind Med. 1999 Jan;35(1):1-8. | Has no available data |  |
| 307 | Szeszenia-Dabrowska N, | 1999 | Int J Occup Med Environ Health. 1998;11(2):171-7. | Not related to chrome |  |
| 308 | Belli S, | 1998 | Epidemiol Prev. 1998 Jan-Mar;22(1):8-11. Italian. | Related to asbestos |  |
| 309 | Magnani C, | 1998 | Occup Environ Med. 1998 Feb;55(2):111-4. | Related to asbestos |  |
| 310 | Weisskopf M, | 1998 | Unfallchirurg. 1998 Mar;101(3):238-41. German. | Not related to chrome |  |
| 311 | van Loon AJ, | 1997 | Occup Environ Med. 1997 Nov;54(11):817-24. | Has no available data |  |
| 312 | Raffn E, | 1998 | Ugeskr Laeger. 1998 Feb 9;160(7):1029-33. Danish. | Related to asbestos |  |
| 313 | Chien SH, | 1997 | Kaohsiung J Med Sci. 1997 Sep;13(9):556-61. | Not related to chrome |  |
| 314 | Lipscomb HJ, | 1997 | Am J Ind Med. 1998 Feb;33(2):131-50. | Not related to chrome |  |
| 315 | Case BW, | 1997 | Environ Health Perspect. 1997 Sep;105 Suppl 5:1113-9. | Not related to chrome |  |
| 316 | Finkelstein MM. | 1997 | Am J Ind Med. 1997 Oct;32(4):341-8. | Related to asbestos |  |
| 317 | Aw TC. | 1997 | Regul Toxicol Pharmacol. 1997 Aug;26(1 Pt 2):S8-S12. | Not related to chrome |  |
| 318 | Holly EA, | 1997 | J Acquir Immune Defic Syndr Hum Retrovirol. 1997 Jul 1;15(3):223-31. | Not related to chrome |  |
| 319 | Stern F, | 1997 | Am J Ind Med. 1997 Jul;32(1):51-65. | Not related to chrome |  |
| 320 | Benke G, | 1997 | Int J Epidemiol. 1997 Jun;26(3):635-42. | Not related to chrome |  |
| 321 | Chiazze L Jr, | 1997 | J Occup Environ Med. 1997 May;39(5):432-41. | Not related to chrome |  |
| 322 | Moulin JJ. | 1997 | Scand J Work Environ Health. 1997 Apr;23(2):104-13. | [A meta-analysis](https://www.ncbi.nlm.nih.gov/pubmed/9167233) |  |
| 323 | van Loon AJ, | 1997 | J Epidemiol Community Health. 1997 Feb;51(1):24-9. | Not related to chrome |  |
| 324 | Mancuso TF. | 1997 | Am J Ind Med. 1997 Feb;31(2):129-39. | Has no available data |  |
| 325 | Szeszenia-Dabrowska N, | 1997 | Med Pr. 1997;48(5):473-83. Polish. | Related to asbestos |  |
| 326 | Dijkstra S, | 1996 | Eur J Surg Oncol. 1996 Dec;22(6):621-6. | Not related to chrome |  |
| 327 | Alexander BH, | 1996 | J Occup Environ Med. 1996 Dec;38(12):1253-8. | Has no available data |  |
| 328 | Harty LC, | 1996 | Cancer Epidemiol Biomarkers Prev. 1996 Dec;5(12):997-1003. | Not related to chrome |  |
| 329 | Manni A, | 1996 | Clin Cancer Res. 1996 Nov;2(11):1901-6. | Not related to chrome |  |
| 330 | Ascoli V, | 1996 | Tumori. 1996 Nov-Dec;82(6):526-32. | Not related to chrome |  |
| 331 | Hansen KS. | 1996 | Am J Ind Med. 1996 Oct;30(4):392-7. | Has no available data |  |
| 332 | Lauritsen JM, | 1996 | Am J Ind Med. 1996 Oct;30(4):383-91. | OR |  |
| 333 | Lammer J, | 1996 | Radiology. 1996 Oct;201(1):167-72. | Not related to chrome |  |
| 334 | Germani D, | 1996 | Med Lav. 1996 Sep-Oct;87(5):371-85. Italian. | Not related to chrome |  |
| 335 | Raffn E, | 1996 | Am J Ind Med. 1996 Sep;30(3):267-72. | Related to asbestos |  |
| 336 | Mikoczy Z, | 1996 | Occup Environ Med. 1996 Jul;53(7):463-7. | OR |  |
| 337 | Ueyama Y, | 1996 | J Oral Maxillofac Surg. 1996 Jul;54(7):858-62; discussion 862-3. | Not related to chrome |  |
| 338 | Raffn E, | 1996 | Occup Environ Med. 1996 Jun;53(6):399-402. | Related to asbestos |  |
| 339 | Fu H, | 1996 | Occup Environ Med. 1996 Jun;53(6):394-8. | [An analysis of two cohorts.](https://www.ncbi.nlm.nih.gov/pubmed/8758034) |  |
| 340 | Rosenman KD, | 1996 | Am J Ind Med. 1996 May;29(5):491-500. | Not related to chrome |  |
| 341 | Schmassmann A, | 1996 | Am J Gastroenterol. 1996 Apr;91(4):654-9. | PCMR |  |
| 342 | Magnani C, | 1996 | Med Lav. 1996 Mar-Apr;87(2):133-46. Italian. | Not related to chrome |  |
| 343 | Itoh T, | 1996 | J UOEH. 1996 Mar 1;18(1):7-18. Japanese. | Has no available data |  |
| 344 | Haentjens P, | 1996 | Bull Cancer. 1995 Nov;82(11):961-70. | Not related to chrome |  |
| 345 | Linet MS, | 1996 | J Occup Environ Med. 1995 Sep;37(9):1127-35. | Has no available data |  |
| 346 | Robinson C, | 1996 | Am J Ind Med. 1995 Jul;28(1):49-70. | Has no available data |  |
| 347 | Finkelstein MM. | 1996 | Am J Ind Med. 1995 Jul;28(1):41-7. | Not related to chrome |  |
| 348 | Magnani C, | 1995 | Occup Environ Med. 1995 Jun;52(6):362-7. | Not related to chrome |  |
| 349 | Marini F, | 1995 | Scand J Work Environ Health. 1995 Feb;21(1):65-8. | Not related to chrome |  |
| 350 | Moulin JJ, | 1995 | Rev Epidemiol Sante Publique. 1995;43(2):107-21. French. | Not related to chrome |  |
| 351 | Deschamps F, | 1995 | Int Arch Occup Environ Health. 1995;67(3):147-52. | Has no available data |  |
| 352 | Dijstra S, | 1994 | Eur J Surg. 1994 Oct;160(10):535-42. | Not related to chrome |  |
| 353 | Park R, | 1994 | Am J Ind Med. 1994 Oct;26(4):449-63. | Not related to chrome |  |
| 354 | Ell C, | 1994 | Am J Gastroenterol. 1994 Sep;89(9):1496-500. | Not related to chrome |  |
| 355 | Langård S. | 1994 | Sci Total Environ. 1994 Jun 6;148(2-3):303-9. Review. | Review |  |
| 356 | Chow WH, | 1994 | J Occup Med. 1994 Jun;36(6):647-51. | Has no available data |  |
| 357 | Rudondy P, | 1994 | Cardiovasc Surg. 1994 Jun;2(3):344-9. | Not related to cancer |  |
| 358 | de Souza AC, | 1994 | Ann Thorac Surg. 1994 Jun;57(6):1573-7; discussion 1577-8. | Not related to chrome |  |
| 359 | Pastides H, | 1994 | Am J Ind Med. 1994 May;25(5):663-75. | OR |  |
| 360 | Pettinari A, | 1994 | Med Lav. 1994 May-Jun;85(3):223-30. Italian. | Has no available data |  |
| 361 | Becker N, | 1994 | Math Biosci. 1994 Apr;120(2):147-63. | Not related to chrome |  |
| 362 | Egan AM, | 1994 | Clin Radiol. 1994 Mar;49(3):162-5. | Has no available data |  |
| 363 | Jakobsson K, | 1994 | Occup Environ Med. 1994 Feb;51(2):95-101. | Has no available data |  |
| 364 | Prionas SD, | 1994 | Int J Radiat Oncol Biol Phys. 1994 Jan 1;28(1):151-62. | Not related to chrome |  |
| 365 | Spanish | 1994 | Arch Bronconeumol. 1994 Jan;30(1):40-8. Spanish. | Not related to chrome |  |
| 366 | Giaroli C, | 1994 | Int Arch Occup Environ Health. 1994;66(1):7-11. | Related to asbestos |  |
| 367 | Jónsson B, | 1994 | Eur Spine J. 1994;3(2):76-83. | Not related to chrome |  |
| 368 | LANGARD, S | 1994 | SCIENCE OF THE TOTAL ENVIRONMENT:148(2-3): 303-309 | Has no available data |  |
| 369 | Danielsen TE, | 1993 | Br J Ind Med. 1993 Dec;50(12):1097-103. | Not related to chrome |  |
| 370 | Andersen A, | 1993 | Am J Epidemiol. 1993 Nov 1;138(9):682-7. | Not related to chrome |  |
| 371 | Teshima T, | 1993 | Radiat Med. 1993 Nov-Dec;11(6):242-6. | Not related to chrome |  |
| 372 | Teshima T, | 1993 | Radiat Med. 1993 Nov-Dec;11(6):237-41. | Not related to chrome |  |
| 373 | Wong O, | 1993 | J Environ Pathol Toxicol Oncol. 1993 Oct-Dec;12(4):171-83. Review. | Not related to chrome |  |
| 374 | Magnani C, | 1993 | Br J Ind Med. 1993 Sep;50(9):779-84. | Related to asbestos |  |
| 375 | Vestbo J. | 1993 | Dan Med Bull. 1993 Mar;40(1):1-16. | Review. |  |
| 376 | Kano K, | 1993 | Int J Epidemiol. 1993 Feb;22(1):16-22. Erratum in: Int J Epidemiol 1993 Aug;22(4):757. | Has no available data |  |
| 377 | Luce D, | 1993 | Int J Cancer. 1993 Jan 21;53(2):224-31. | Has no available data |  |
| 378 | Raffn E, | 1993 | Br J Ind Med. 1993 Jan;50(1):85-9. | Related to asbestos |  |
| 379 | Rosenman KD, | 1993 | Am J Ind Med. 1993 Jan;23(1):125-34. | Has no available data |  |
| 380 | Korallus U, | 1993 | Int Arch Occup Environ Health. 1993;65(3):171-8. | Has no available data |  |
| 381 | Fletcher AC, | 1993 | Int J Epidemiol. 1993;22 Suppl 2:S29-35. | Not related to chrome |  |
| 382 | Gérin M, | 1993 | Int J Epidemiol. 1993;22 Suppl 2:S22-8. | Has no available data |  |
| 383 | Gurvich EB, | 1993 | Med Tr Prom Ekol. 1993;(5-6):4-6. Russian. | Not related to chrome |  |
| 384 | Lugmayr H, | 1993 | AJR Am J Roentgenol. 1992 Nov;159(5):1091-4. | Not related to chrome |  |
| 385 | Lee MJ, | 1992 | J Vasc Interv Radiol. 1992 Nov;3(4):665-71. | Not related to chrome |  |
| 386 | Cheng WN, | 1992 | Environ Res. 1992 Oct;59(1):271-8. | Related to asbestos |  |
| 387 | Kromhout H, | 1992 | Am J Epidemiol. 1992 Sep 15;136(6):698-711. | Not related to chrome |  |
| 388 | Johansson L, | 1992 | Br J Ind Med. 1992 Sep;49(9):626-30. | Related to asbestos |  |
| 389 | Tulchinsky TH, | 1992 | Isr J Med Sci. 1992 Aug-Sep;28(8-9):543-7. | Related to asbestos |  |
| 390 | Dinehart SM, | 1992 | J Dermatol Surg Oncol. 1992 Jul;18(7):560-6. | Not related to chrome |  |
| 391 | Bonde JP, | 1992 | Scand J Work Environ Health. 1992 Jun;18(3):169-77. | Not related to chrome |  |
| 392 | Verma DK, | 1992 | Am Ind Hyg Assoc J. 1992 May;53(5):317-24. | Not related to chrome |  |
| 393 | Gordon RL, | 1992 | Radiology. 1992 Mar;182(3):697-701. | Not related to chrome |  |
| 394 | Rosen RH, | 1992 | Australas J Dermatol. 1992;33(1):1-10. | Not related to chrome |  |
| 395 | Merler E, | 1992 | Ann Ist Super Sanita. 1992;28(1):133-46. Review. Italian. | Review |  |
| 396 | Zaridze DG, | 1992 | Vopr Onkol. 1992;38(9):1066-73. Russian. | Not related to chrome |  |
| 397 | Gurvich EB, | 1992 | Gig Tr Prof Zabol. 1992;(7):17-20. Russian. | Not related to chrome |  |
| 398 | Finkelstein MM. | 1991 | Ann N Y Acad Sci. 1991 Dec 31;643:85-9. No abstract available. | Related to asbestos |  |
| 399 | Vestbo J, | 1991 | Br J Ind Med. 1991 Dec;48(12):803-7. | RR |  |
| 400 | Papazian MR, | 1991 | J Oral Maxillofac Surg. 1991 Oct;49(10):1055-9; discussion 1059-60. | Not related to chrome |  |
| 401 | Dubousset J, | 1991 | Clin Orthop Relat Res. 1991 Sep;(270):52-9. | Not related to chrome |  |
| 402 | Song HY, | 1991 | Radiology. 1991 Aug;180(2):349-54. | Not related to chrome |  |
| 403 | Gullane PJ. | 1991 | Laryngoscope. 1991 Jun;101(6 Pt 2 Suppl 54):1-24. | Not related to chrome |  |
| 404 | Hughes JM, | 1991 | Br J Ind Med. 1991 Apr;48(4):229-33. | Not related to chrome |  |
| 405 | Simonato L, | 1991 | Br J Ind Med. 1991 Mar;48(3):145-54. | Not related to chrome |  |
| 406 | Weinstock MA, | 1991 | Am J Epidemiol. 1991 Feb 1;133(3):240-5. | Not related to chrome |  |
| 407 | Steenland K, | 1991 | Am J Epidemiol. 1991 Feb 1;133(3):220-9. | Has no available data |  |
| 408 | Botta M, | 1991 | Cancer Detect Prev. 1991;15(6):445-7. | Related to asbestos |  |
| 409 | Chen JG. | 1990 | Zhonghua Yu Fang Yi Xue Za Zhi. 1990 Nov;24(6):328-31. Chinese. | Not related to chrome |  |
| 410 | Albin M, | 1990 | Br J Ind Med. 1990 Sep;47(9):602-10. | Related to asbestos |  |
| 411 | Neuberger M, | 1990 | Br J Ind Med. 1990 Sep;47(9):615-20. | Related to asbestos |  |
| 412 | Bonassi S, | 1990 | Epidemiol Prev. 1990 Sep;12(44):25-30. Italian. | Has no available data |  |
| 413 | Moulin JJ, | 1990 | Br J Ind Med. 1990 Aug;47(8):537-43. | Duplicated data |  |
| 414 | Seniori Costantini A, | 1990 | Med Lav. 1990 May-Jun;81(3):184-211. Italian. | Not related to chrome |  |
| 415 | Yazawa Y, | 1990 | Clin Orthop Relat Res. 1990 Feb;(251):213-9. | Not related to chrome |  |
| 416 | Langård S. | 1990 | Am J Ind Med. 1990;17(2):189-215. Review. | Review |  |
| 417 | Magnani C, | 1989 | Acta Otorhinolaryngol Ital. 1989 Sep-Oct;9(5):511-9. Review. Italian. | Review |  |
| 418 | Merlo F, | 1989 | J UOEH. 1989 Mar 20;11 Suppl:302-15. | Not related to chrome |  |
| 419 | Centers for Disease Control (CDC). | 1989 | MMWR Morb Mortal Wkly Rep. 1989 Feb 24;38(7):105-6, 111-4. | Not related to chrome |  |
| 420 | Raffn E, | 1989 | Br J Ind Med. 1989 Feb;46(2):90-6. | Related to asbestos |  |
| 421 | Gubéran E, | 1989 | Br J Ind Med. 1989 Jan;46(1):16-23. | Not related to chrome |  |
| 422 | Hayes RB, | 1989 | Am J Ind Med. 1989;16(2):127-33. | Has no available data |  |
| 423 | Hull CJ, | 1989 | Am J Ind Med. 1989;16(1):103-12 | Not related to chrome |  |
| 424 | de Marco R, | 1988 | Med Lav. 1988 Sep-Oct;79(5):368-76. Italian. | Not related to chrome |  |
| 425 | Tola S, | 1988 | Br J Ind Med. 1988 Apr;45(4):209-18. | Not exposed to chrome |  |
| 426 | Dancuart F, | 1988 | Int J Radiat Oncol Biol Phys. 1988 Apr;14(4):745-9. | Not related to chrome |  |
| 427 | Teta MJ, | 1988 | Am J Epidemiol. 1988 Mar;127(3):540-51. | Not related to chrome |  |
| 428 | Rinsky RA, | 1988 | Am J Epidemiol. 1988 Jan;127(1):55-64. | Not related to chrome |  |
| 429 | TOLA, S | 1988 | BRITISH JOURNAL OF INDUSTRIAL MEDICINE : 45(4):209-218  APR 1988 | Has no available data |  |
| 430 | Magnani C, | 1987 | Med Lav. 1987 Nov-Dec;78(6):441-53. Italian. No abstract available. | Related to asbestos |  |
| 431 | Ackermann G, | 1987 | Oral Surg Oral Med Oral Pathol. 1987 Sep;64(3):308-12. | Not related to chrome |  |
| 432 | Stern FB, | 1987 | Scand J Work Environ Health. 1987 Apr;13(2):108-17. | Duplicated data |  |
| 433 | Sorahan T, | 1987 | Br J Ind Med. 1987 Apr;44(4):250-8. | Not related to chrome |  |
| 434 | Guay D, | 1987 | Am J Ind Med. 1987;12(2):181-93. | Not related to chrome |  |
| 435 | Niederle B, | 1986 | Surgery. 1986 Dec;100(6):1088-97. | Not related to chrome |  |
| 436 | Gardner MJ, | 1986 | Br J Ind Med. 1986 Nov;43(11):726-32. | Related to asbestos |  |
| 437 | Amandus HE. | 1986 | Br J Ind Med. 1986 Aug;43(8):526-8. | Not related to chrome |  |
| 438 | Coggon D, | 1986 | Br J Ind Med. 1986 May;43(5):332-8. | Not related to chrome |  |
| 439 | Steenland K, | 1986 | J Chronic Dis. 1986;39(4):287-94. | Has no available data |  |
| 440 | Goldsmith JR. | 1986 | Am J Ind Med. 1986;10(5-6):543-52. | Related to asbestos |  |
| 441 | Dorr LD, | 1986 | J Arthroplasty. 1986;1(1):21-8. | Not related to chrome |  |
| 442 | Sweeney MH, | 1985 | Scand J Work Environ Health. 1985 Aug;11(4):257-64. | Not related to chrome |  |
| 443 | Neuberger M | 1985 | Zentralbl Bakteriol Mikrobiol Hyg B. 1985 Jun;181(1-2):81-6. German. | Related to asbestos |  |
| 444 | Ohlson CG, | 1985 | Br J Ind Med. 1985 Jun;42(6):397-402. | Related to asbestos |  |
| 445 | Becker N, | 1985 | Scand J Work Environ Health. 1985 Apr;11(2):75-82. | Has no available data |  |
| 446 | Finkelstein MM, | 1984 | Am Rev Respir Dis. 1984 Jan;129(1):17-22. | Related to asbestos |  |
| 447 | Simpson SG, | 1983 | Arch Environ Health. 1983 Jul-Aug;38(4):248-51. | Not related to chrome |  |
| 448 | Mel'nikov RA, | 1983 | Vestn Khir Im I I Grek. 1983 Mar;130(3):66-72. Russian. | Not related to chrome |  |
| 449 | Langård S, | 1983 | Br J Ind Med. 1983 Feb;40(1):71-4. | Not related to chrome |  |
| 450 | Frentzel-Beyme R. | 1983 | J Cancer Res Clin Oncol. 1983;105(2):183-8. | Has no available data |  |
| 451 | Thomas HF, | 1982 | Br J Ind Med. 1982 Aug;39(3):273-6. | Related to asbestos |  |
| 452 | Delzell E, | 1982 | J Occup Med. 1982 Jul;24(7):539-45. | Not related to chrome |  |
| 453 | Sheffet A, | 1982 | Arch Environ Health. 1982 Jan-Feb;37(1):44-52. | Not related to chrome |  |
| 454 | Alderson MR, | 1981 | Br J Ind Med. 1981 May;38(2):117-24. | Not related to chrome |  |
| 455 | Sjögren B. | 1980 | Scand J Work Environ Health. 1980 Sep;6(3):197-200. | Not related to chrome |  |
| 456 | Dalager NA, | 1980 | J Occup Med. 1980 Jan;22(1):25-9. | Not related to chrome |  |
| 457 | Enterline PE, | 1980 | IARC Sci Publ. 1980;(30):973-7. | Not related to chrome |  |
| 458 | Haider M, | 1980 | IARC Sci Publ. 1980;(30):973-7. | Related to asbestos |  |
| 459 | Hughes J, | 1980 | IARC Sci Publ. 1980;(30):627-35. | Related to asbestos |  |
| 460 | Weill H, | 1979 | Am Rev Respir Dis. 1979 Aug;120(2):345-54. | Related to asbestos |  |
| 461 | Puntoni R, | 1979 | Ann N Y Acad Sci. 1979;330:353-77. | Not related to chrome |  |
| 462 | Harrington JM, | 1978 | Am J Epidemiol. 1978 Feb;107(2):96-103. | Related to asbestos |  |
| 463 | Royle H. | 1975 | Environ Res. 1975 Aug;10(1):39-53. | Not related to chrome |  |
| 464 | Langård S, | 1975 | Br J Ind Med. 1975 Feb;32(1):62-5. | Not related to chrome |  |
| 465 | Enterline PE. | 1974 | J Occup Med. 1974 Aug;16(8):523-6. | Not related to chrome |  |
| 466 | Dunn JE Jr, | 1968 | Arch Environ Health. 1968 Jul;17(1):71-6. | Not related to chrome |  |
